# Innate immune training of osteoclastogenesis promotes inflammatory bone loss in mice

**DOI:** 10.1016/j.devcel.2025.02.001

**Published:** 2025-02-27

**Authors:** Nora Haacke, Hui Wang, Shu Yan, Marko Barovic, Xiaofei Li, Kosuke Nagai, Adelina Botezatu, Aikaterini Hatzioannou, Bettina Gercken, Giulia Trimaglio, Anisha U. Shah, Jun Wang, Ling Ye, Mangesh T. Jaykar, Martina Rauner, Ben Wielockx, Kyoung-Jin Chung, Mihai G. Netea, Lydia Kalafati, George Hajishengallis, Triantafyllos Chavakis

**Affiliations:** 1 Institute for Clinical Chemistry and Laboratory Medicine, Faculty of Medicine, TU Dresden, 01307 Dresden, Germany; 2 Department of Basic and Translational Sciences, Laboratory of Innate Immunity and Inflammation, Penn Dental Medicine, University of Pennsylvania, Philadelphia, PA 19104, USA; 3 State Key Laboratory of Oral Diseases, National Center for Stomatology, National Clinical Research Center for Oral Diseases, Department of Periodontics, West China Hospital of Stomatology, Sichuan University, Chengdu 610041, Sichuan, China; 4 National Center for Tumor Diseases, Partner Site Dresden, 01307 Dresden, Germany; 5 Sheng Yushou Center of Cell Biology and Immunology, School of Life Sciences and Biotechnology, Shanghai Jiao Tong University, Shanghai 200240, China; 6 State Key Laboratory of Oral Diseases, National Center for Stomatology, National Clinical Research Center for Oral Diseases, Department of Endodontics, West China Hospital of Stomatology, Sichuan University, Chengdu 610041, Sichuan, China; 7 Department of Medicine III & Center for Healthy Aging, Faculty of Medicine, TU Dresden, 01307 Dresden, Germany; 8 Department of Internal Medicine and Radboud Center for Infectious Diseases, Radboud University Medical Center, 6525 XZ Nijmegen, the Netherlands; 9 Department of Immunology and Metabolism, Life and Medical Sciences Institute, University of Bonn, 53115 Bonn, Germany; 10 Institute of Radiopharmaceutical Cancer Research, Helmholtz-Zentrum Dresden-Rossendorf, 01328 Dresden, Germany; 11 Paul Langerhans Institute Dresden of the Helmholtz Center Munich, University Hospital and Faculty of Medicine, TU Dresden, 01307 Dresden, Germany; 12 German Center for Diabetes Research (DZD), 85764 Neuherberg, Germany

## Abstract

We previously demonstrated that long-term trained immunity (TRIM) involves adaptations that imprint innate immune memory in long-lived myelopoiesis precursors and their progeny, monocytes/macrophages and neutrophils, which thereby acquire enhanced responsiveness to future challenges. Here, we show that a distinct component of myeloid biology, osteoclastogenesis, can also undergo innate immune training. Indeed, β-glucan-induced TRIM was associated with an increased osteoclastogenesis bias in the bone marrow and an expansion of monocytes/osteoclast progenitors in the periphery, resulting in aggravated severity of experimental periodontitis and arthritis. In the setting of trained inflammatory osteoclastogenesis, we observed transcriptomic rewiring in synovial myeloid cells of arthritic mice, featuring prominent upregulation of the transcription factor melanogenesis-associated transcription factor (MITF). Adoptive transfer of splenic monocytes from β-glucan-trained mice to naive recipients exacerbated arthritis in the latter in a strictly MITF-dependent manner. Our findings establish trained osteoclastogenesis as a maladaptive component of TRIM and potentially provide therapeutic targets in inflammatory bone loss disorders.

## Graphical abstract

**Graphical abstract:**
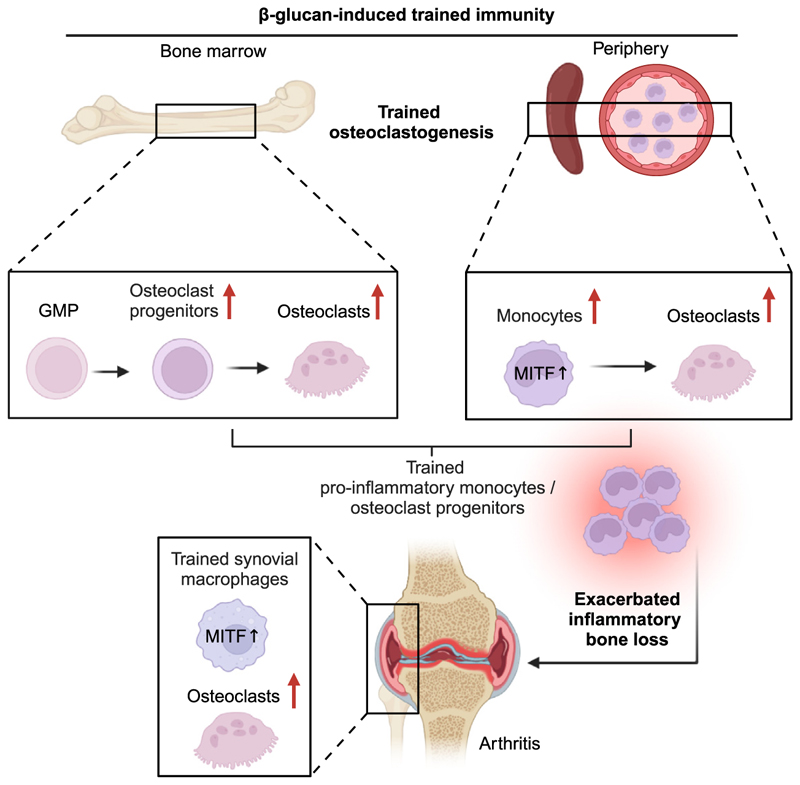

## Introduction

Disorders associated with inflammatory bone loss include periodontitis and inflammatory arthritis.^1–3^ In periodontitis and arthritis, a complex network of inflammatory cytokines and immune cells operates together with osteoclasts, the professional bone-resorbing cells, to promote gingival or synovial inflammation and bone loss.^1,2,4,5^

Osteoclasts are large, multinucleated bone-resorbing cells that derive from the myeloid lineage.^4,6^ Osteoclastogenesis is facilitated by the synergistic actions of macrophage colony stimulating factor (M-CSF), which binds to the CSF-1 receptor (CSF1R; CD115), with receptor activator of nuclear factor κ B (NF-κ B) ligand (RANKL).^6,7^ However, alternative pathways of osteoclast differentiation have been described in inflammation.^8^ Osteoclasts share a common progenitor with monocytes/macrophages and dendritic cells in the bone marrow (BM), which lies downstream of the granulocyte-macrophage progenitors (GMPs).^9–11^ Aside from these BM progenitors that produce osteoclasts mainly operating in homeostatic bone remodeling, several other cell types have been identified as potential osteoclast progenitors (OCPs), especially during inflammation.^4,12–16^ Cells harboring OCP potential include monocytes and macrophages.^4,12,13,17^ For example, classical (CD11b^+^CD115^+^Ly6C^high^) monocytes may contain OCP activity and generate osteoclasts.^13,16^ In the inflamed arthritic synovium, monocyte-derived macrophage subpopulations may also give rise to osteoclasts.^12^ Recruitment of OCPs from the BM, the spleen, or the blood, and their differentiation to osteoclasts, contributes to bone resorption in inflammatory bone loss disorders. In summary, OCPs display large heterogeneity, especially under conditions of inflammation-associated bone loss.^18^

In arthritis and periodontitis, cytokines, such as interleukin (IL)-17, tumor necrosis factor (TNF), IL-6, and IL-1β, produced by diverse cells of innate and adaptive immunity, contribute to synovial or gingival inflammation and amplify bone erosion via direct or indirect stimulation of osteoclast generation and activation, in part by upregulating expression of RANKL in different cell types, including synovial fibroblasts in arthritis.^2,19–23^

Trained immunity (TRIM), the enhanced immune response consequent to induction of innate immune memory, has recently emerged as a key principle in the long-term regulation of inflammatory responses.^24–26^ Certain pathogen-derived agonists, such as fungal β-glucan, or vaccines endow myeloid cells with enhanced inflammatory preparedness via sustained metabolic, epigenetic, and transcriptomic rewiring, which, in turn, promotes increased responses to future challenges.^24^ We and others have previously established that the prolonged actions of TRIM, despite the short lifetime of myeloid cells in the circulation, are mediated by rewiring of long-lived BM progenitors, including hematopoietic stem and progenitor cells (HSPCs).^27–30^ Although TRIM can exert beneficial actions by protecting against infections^31–34^ and cancer,^30,35^ inflammatory memory imprinted in BM hematopoietic progenitors may also contribute to inflammatory diseases and the comorbidity between inflammatory bone loss disorders.^36–40^ However, whether osteoclastogenesis itself is subject to innate immune training under these conditions is entirely unknown.

This is of particular importance in the context of the potent anti-cancer effects of β-glucan-induced TRIM.^30^ Indeed, the use of β-glucan preparations in clinical trials for cancer immunotherapies,^41,42^ often in combination with immune checkpoint inhibitors,^42^ may require caution given that inflammatory bone loss is a frequent side effect of cancer immunotherapies, particularly of immune checkpoint inhibitors.^43,44^ Hence, understanding the potential role of β-glucan-induced TRIM in osteoclastogenesis and inflammatory bone loss is imperative. Here, we demonstrate that innate immune training of inflammatory osteoclastogenesis is an additional facet of TRIM that amplifies inflammatory bone loss.

## Results

### TRIM promotes inflammatory bone loss in experimental periodontitis and arthritis

**Figure 1:**
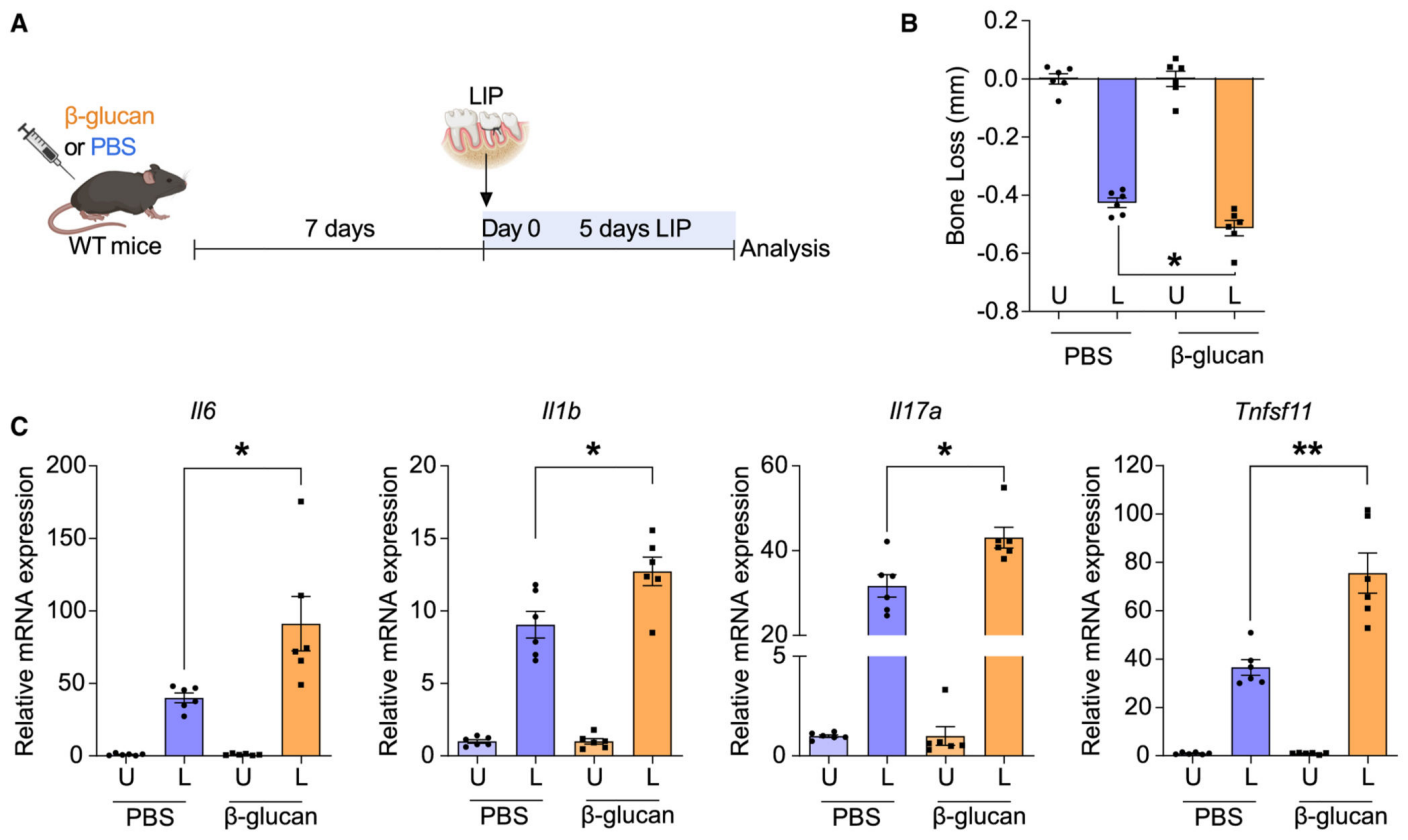
TRIM promoted inflammatory bone loss in LIP (A) Mice were pre-treated with β-glucan or PBS-control. After 7 days, periodontal bone loss was induced for 5 days in both groups by ligating a maxillary second molar and leaving the contralateral tooth unligated. (B) Periodontal bone loss 5 days after LIP induction (*n* = 6 mice per group). (C) Relative mRNA expression of the indicated molecules in gingival tissues, harvested on day 5 of LIP (*n* = 6 mice per group). Results are presented as fold change in the transcript levels in ligated sites relative to those of corresponding unligated sites, which were assigned a value of 1. Data are mean ± SEM; *p < 0.05, **p < 0.01. Unpaired t test (B and C) except for *Il17a* in (C) (Mann-Whitney U test). U, unligated; L, ligated.

To explore the role of TRIM in inflammatory bone loss pathologies, we employed mouse models of experimental periodontitis and arthritis. We first pre-treated wild-type (WT) mice with an intraperitoneal (i.p.) injection of the prototype agonist of TRIM, β-glucan,^27^ or PBS as control. 7 days thereafter, β-glucantreated (trained) or control-treated (non-trained) mice were subjected to ligature-induced periodontitis (LIP) by ligating the maxillary second molar, with the contralateral tooth kept unligated to serve as baseline control (Figure 1A); periodontitis was analyzed 5 days later. Compared with untrained controls, trained mice exhibited significantly increased periodontal bone loss at the ligated sites (Figure 1B). Expression of proinflammatory genes (*Il6, Il1b*, and *Il17a*) was increased in the gingival tissue of trained mice, compared with non-trained controls, 5 days after LIP induction (Figure 1C). Additionally, *Tnfsf11*, encoding for the cardinal osteoclastogenic factor RANKL, was up-regulated in the gingival tissue of LIP-subjected trained mice (Figure 1C). Therefore, β-glucan-induced TRIM exacerbates inflammatory bone loss upon application of a secondary stimulus (LIP).

**Figure 2:**
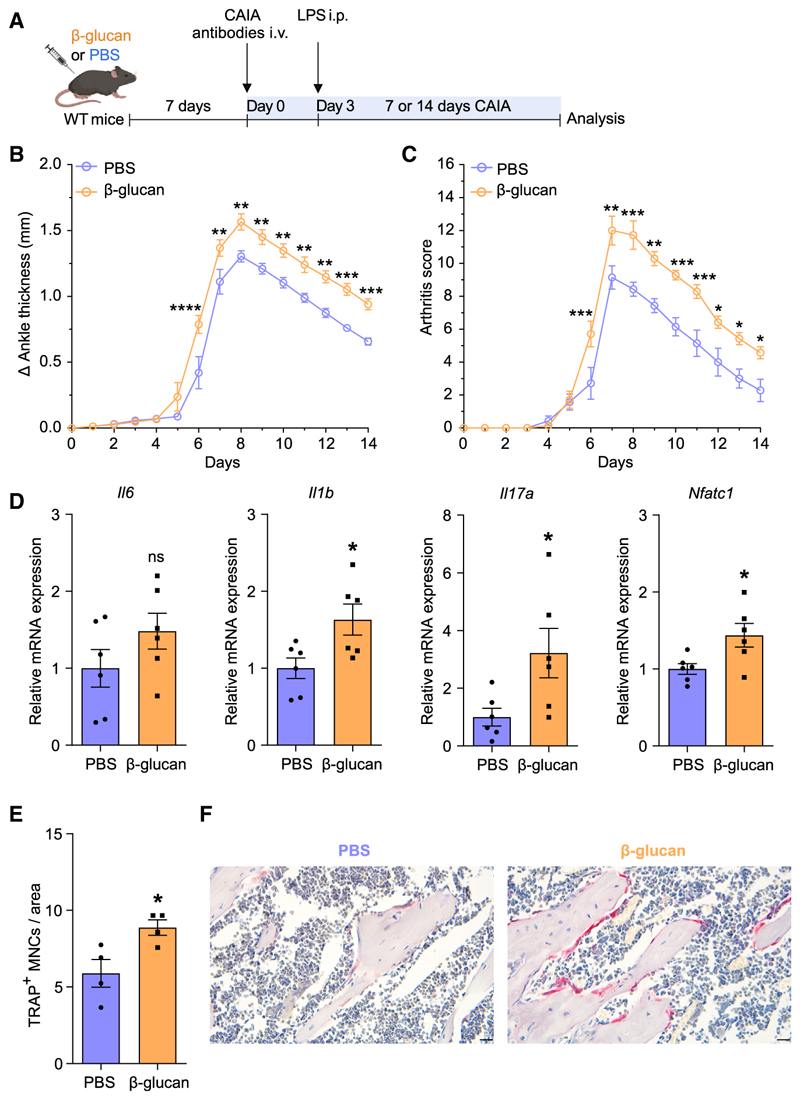
Innate immune training with β-glucan enhanced disease severity in CAIA (A) Mice were pre-treated with β-glucan or PBS-control. After 7 days, both groups of mice were subjected to the CAIA model. (B and C) The difference in ankle thickness, i.e., Delta (Δ) ankle thickness (B) and the arthritis score (C) are shown (*n* = 7 mice per group). (D) Relative mRNA expression of the indicated molecules in knee joints, harvested on day 7 of the CAIA model (*n* = 6 mice per group). Results are presented relative to those of the PBS-control group, set as 1. (E and F) Histology analysis (TRAP staining) of knee joints (harvested on day 7 of the CAIA model). (E) Quantification of TRAP^+^ MNCs per area (*n* = 4 mice per group) and (F) representative TRAP-stained images. Scale bars, 20 μm. Data are mean ± SEM; ns, non-significant; *p < 0.05; **p < 0.01; ***p < 0.001; ****p < 0.0001. Two-way ANOVA with repeated-measures and Sidak’s post-test for comparison with PBS group (B and C); unpaired t test (D and E). See also Figure S1.

We next investigated the relevance of these findings in experimental arthritis. Mice were subjected to collagen-antibody-induced arthritis (CAIA) 7 days after TRIM induction by a single i.p. injection with β-glucan or PBS administration as control (Figure 2A). Mice pre-treated with β-glucan developed more severe arthritis compared with untrained controls, as shown by increased ankle thickness and arthritis score (Figures 2B and 2C). Gene expression analysis of the synovium 7 days after CAIA induction revealed up-regulation of inflammatory cytokines *Il1b* and *Il17a* in trained mice compared with non-trained controls, indicating increased inflammatory activity in the joints following TRIM induction (Figure 2D). Expression of *Nfatc1*, a master transcription factor of osteoclastogenesis,^2,45,46^ was up-regulated in the joints of trained mice (Figure 2D). Gene expression analysis from synovial myeloid cells (CD11b^+^) and stromal cells (CD45^-^) of arthritic mice pre-treated with β-glucan or PBS revealed that the expression of inflammatory cytokines was significantly increased in CD11b^+^ but not in CD45^−^ cells due to TRIM induction (Figures S1A and S1B). Thus, synovial myeloid cells, rather than stromal cells, are targeted by TRIM for enhanced cytokine responses. Additionally, *Nfatc1* expression was increased in synovial CD11b^+^ myeloid cells from trained arthritic mice as compared with those from untrained mice (Figure S1A). We then performed tartrate-resistant acid phosphatase (TRAP) staining in the knee joints to assess whether β-glucan-induced TRIM affected osteoclastic activity in arthritis. There was no significant difference in the numbers of osteoclasts (TRAP^+^ multinucleated cells [MNCs]) in the knee joints 7 days after mouse pre-treatment with β-glucan or PBS without subsequent arthritis induction (Figure S1C). In contrast, osteoclast numbers were significantly increased in trained mice 7 days after CAIA induction compared with untrained PBS-control mice (Figures 2E and 2F). Hence, TRIM induction led to increased osteoclastogenesis in the joints only upon a secondary challenge (arthritis), consistent with the TRIM concept.

**Figure 3:**
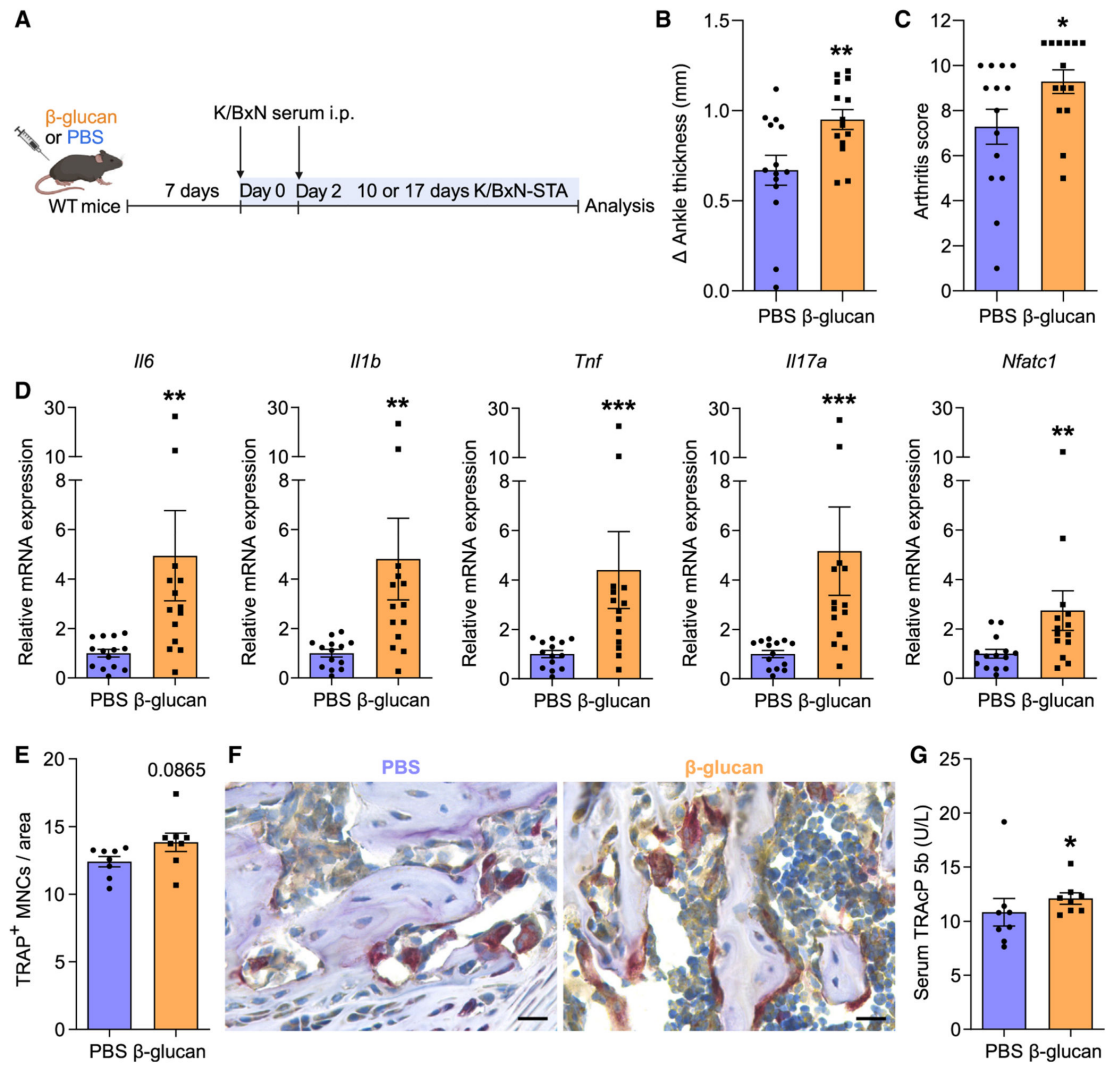
Induction of TRIM exacerbated K/BxN-STA disease (A) Mice were pre-treated with β-glucan or PBS-control. After 7 days, both groups of mice were subjected to K/BxN-STA. (B and C) The difference in ankle thickness, i.e., Delta (Δ) ankle thickness (B) and the arthritis score (C) at the peak of the disease (day 10 after K/BxN-STA induction) are shown. Data are from two independent experiments (*n* = 14 mice per group). (D) Relative mRNA expression of the indicated molecules in knee joints, harvested on day 17 of the K/BxN-STA model. Results are presented relative to those of the PBS-control group, set as 1. Data are from two independent experiments (*n* = 14 mice per group). (E and F) Knee joints (harvested on day 10 of the K/BxN-STA model) were processed for TRAP staining. (E) Quantification of TRAP+ MNCs per area (*n* = 8 mice per group). (F) Representative TRAP-stained images. Scale bars, 20 μm. (G) Concentration of TRAcP 5b in the serum on day 10 of the K/BxN-STA model (*n* = 8 mice per group). Data are mean ± SEM; *p < 0.05, **p < 0.01, ***p < 0.001. Unpaired t test (B and E) or Mann-Whitney U test (C, D, and G). See also Figure S2.

To consolidate the notion that β-glucan-induced TRIM promotes arthritis, we employed an independent model, namely the K/BxN serum transfer arthritis model (K/BxN-STA). Mice were similarly treated with β-glucan or PBS 7 days before injecting K/BxN serum to trigger arthritis (Figure 3A). Consistent with our previous results in LIP and CAIA, training with β-glucan aggravated arthritis, as demonstrated by increased ankle joint thickness and clinical arthritis score at the peak of the disease (Figures 3B and 3C). Enhanced expression of proinflammatory cytokines *Il6, Il1b, Tnf*, and *Il17a* was observed in the synovium of arthritic mice that were trained with β-glucan (Figure 3D). In trained arthritic mice, synovial *Nfatc1* expression was upregulated compared with untrained arthritic mice (Figure 3D), consistent with the findings from the CAIA model. Despite the increased inflammatory signaling in the synovium of trained mice subjected to K/BxN-STA, the frequencies of synovial myeloid cells, including neutrophils (CD11b^+^Ly6G^+^), monocytes (CD11b^+^Ly6G^−^Ly6C^+^), and macrophages (CD11b^+^Ly6G^−^F4/80^+^), were comparable between trained and untrained mice at different time intervals (5, 10, and 17 days), as assessed by flow cytometry (Figures S2A–S2D). Hence, the enhanced proinflammatory phenotype observed in trained mice subjected to K/BxN-STA is associated with qualitative alterations (higher in-flammatory gene expression) rather than quantitative changes in myeloid cell populations. This is consistent with previous observations that TRIM induction is often associated with qualitative rather than quantitative changes.^30^

Histological analysis revealed that osteoclast numbers in the knee joints tended to be higher in β-glucan-trained arthritic mice compared with non-trained arthritic mice (Figures 3E and 3F).

As alluded to above, TRIM may not necessarily lead to quantitative changes^30^; we thus assessed whether there was higher osteoclastic activity in the K/BxN-STA model due to TRIM induction. To this end, we measured the serum levels of TRAP form 5b (TRAcP 5b), a marker of osteoclastic activity and bone resorption. We found higher TRAcP 5b concentrations 10 days after arthritis induction in the K/BxN-STA model in β-glucan-trained compared with untrained mice (Figure 3G), indicating increased osteoclastic activity in K/BxN-STA arthritic mice due to TRIM induction.

Together, β-glucan-induced TRIM exacerbates inflammation, osteoclastic activity, and disease severity in three different mouse models of inflammatory bone loss (LIP, CAIA, and K/BxN-STA).

### Enhanced inflammatory osteoclastogenesis upon β-glucan-induced TRIM

Inflammatory bone loss is influenced not only by inflammation but also by enhanced development and activation of osteoclasts that drive bone erosion.^2^ Whereas it is well established that β-glucan can train innate immune cells,^24,27,30^ the influence, if any, of β-glucan-induced TRIM on osteoclastogenesis has not been explored during inflammatory bone loss. As TRIM is initiated by functional alterations in BM progenitors,^27,36^ we hypothesized that β-glucan-induced innate immune training might have an impact on BM OCPs as well.

**Figure 4:**
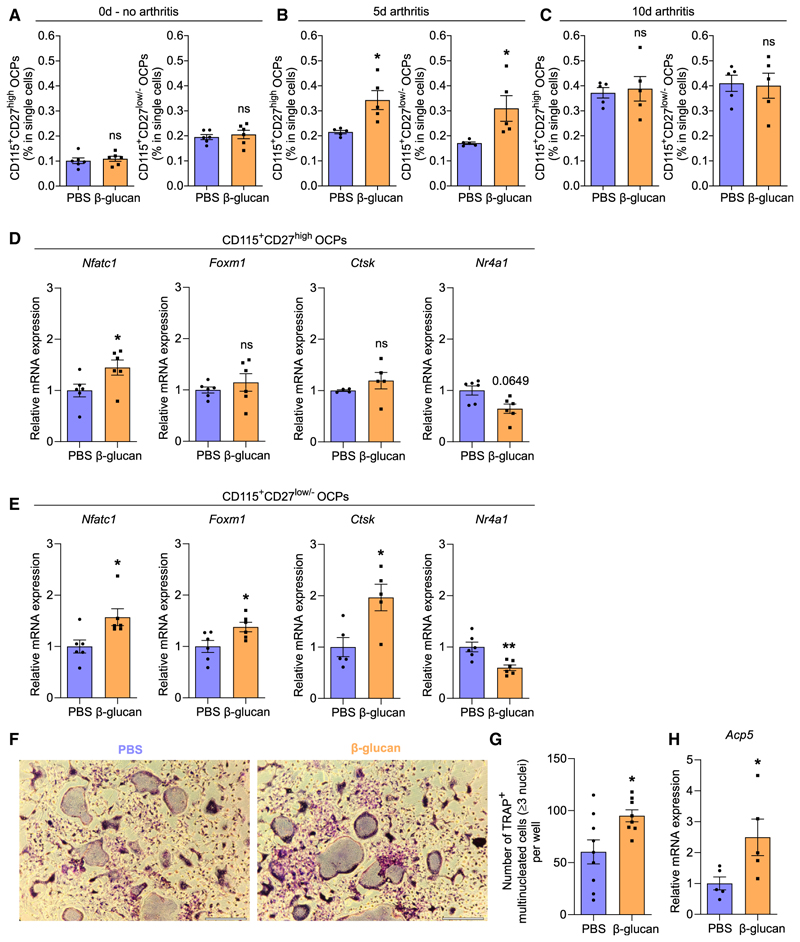
TRIM induced by β-glucan increased BM osteoclastogenesis (A–C) Mice were pre-treated with β-glucan or PBS-control. After 7 days, both groups of mice were subjected to the K/BxN-STA model or not. Flow-cytometric analysis of CD115^+^CD27^high^ and CD115^+^CD27^low/−^ OCPs from BM was performed (A) at day 0 (no arthritis, *n* = 6 mice per group) or (B) at day 5 (*n* = 5 mice per group) and (C) at day 10 (*n* = 5 mice per group) after K/BxN-STA induction. Frequencies of CD115^+^CD27^high^ OCPs (B220^-^c-Kit^+^CD11b^low/−^CD115^+^CD27^high^) and CD115^+^CD27^low/−^ OCPs (B220^-^c-Kit^+^CD11b^low/−^CD115^+^CD27^low/−^) are shown. (D and E) Relative mRNA expression of the indicated molecules from sorted BM CD115^+^CD27^high^ OCPs (D) and CD115^+^CD27^low/−^ OCPs (E) 7 days after β-glucan or PBS treatment (*n* = 4–6 mice per group). Results are presented relative to those of the PBS-control group, set as 1. (F–H) Mice were pre-treated with β-glucan or PBS-control. After 7 days, BM cells were incubated with M-CSF for 3 days followed by another 5 days together with RANKL. Cells were stained for TRAP (F displays representative images; scale bars, 300 μm) to count all mature osteoclasts in the culture wells, defined as TRAP^+^ MNCs with ≥3 nuclei. (G) Quantification of osteoclasts; data are from two independent experiments (in total cultures from *n* = 8–9 mice per group). (H) Relative mRNA expression of *Acp5* from cultured osteoclasts (cultures from *n* = 5 mice per group). Results are presented relative to those of the PBS-control group, set as 1. Data are mean ± SEM; ns, non-significant; *p < 0.05; **p < 0.01. Unpaired t test (A–E, G, and H), except for *Nr4a1* in (D) and *Nfatc1* in (E), in which cases Mann-Whitney U test was used. See also Figure S3.

In the BM of adult mice, the previously described CD115^+^ CD27^high^ and the more committed CD115^+^CD27^low/−^ OCP populations serve as the primary OCPs giving rise to osteoclasts under homeostasis.^4,9^ Both CD115^+^CD27^high^ and CD115^+^ CD27^low/−^ OCPs lie downstream of GMPs, which are important cellular targets of TRIM.^27,28,30^ Flow cytometry was conducted for CD115^+^CD27^high^ OCPs (B220^-^c-Kit^+^CD11b^low/−^CD115^+^ CD27^high^) and CD115^+^CD27^low/−^ OCPs (B220^-^c-Kit^+^CD11b^low/−^ CD115^+^CD27^low/−^) (Figure S3A) in the BM of mice trained with β-glucan or treated with PBS-control for 7 days or mice pretreated with β-glucan or PBS for 7 days and additionally subjected to K/BxN-STA for another 5 or 10 days. Although β-glucan-induced TRIM alone (without subsequent arthritis induction) did not affect the frequencies of CD115^+^CD27^high^ and CD115^+^CD27^low/−^ OCPs in the BM (Figure 4A), 5 days after K/BxN-STA induction, both populations significantly expanded in β-glucan-trained mice compared with non-trained mice (Figure 4B). This effect diminished 10 days after arthritis induction (Figure 4C). Thus, β-glucan-induced TRIM modulates CD115^+^CD27^high^ and CD115^+^ CD27^low/−^ OCPs in a manner that enables them to expand (relative to their counter-parts from untrained mice) upon a secondary challenge (arthritis). To provide additional evidence for innate immune training of osteoclastogenesis, we assessed qualitative changes in OCPs by analyzing expression of osteoclastrelated genes and transcription factors in both CD115^+^CD27^high^ and CD115^+^ CD27^low/−^ OCPs. Although β-glucan-mediated TRIM induction (without secondary arthritis challenge) did not affect CD115^+^CD27^high^ and CD115^+^CD27^low/−^ OCP abundance (Figure 4A), *Nfatc1* expression was significantly upregulated in both populations upon TRIM induction. *Foxm1*,a transcription factor promoting osteoclast development,^12^ and *Ctsk*, an established late-stage osteoclast marker gene,^6^ were selectively upregulated in CD115^+^CD27^low/−^ OCPs, the population bearing elevated OCP potential,^9^ in trained mice relative to untrained controls. No changes in expression of aforementioned factors were observed in CD115^+^CD27^high^ OCPs. Expression of *Nr4a1*, a negative regulator of osteoclast development,^47,48^ was downregulated in CD115^+^CD27^low/−^ OCPs in trained mice compared with untrained controls (Figures 4D and 4E). Expression of other osteoclastogenesis regulators (*Oscar, Mafb*, and *Irf8*) in both populations was comparable between trained and untrained mice (Figures S3B and S3C).

Hence, BM OCPs, especially the CD115^+^CD27^low/−^ OCPs that have higher OCP potential, display an increased bias toward osteoclastogenesis upon β-glucan-mediated TRIM induction. To further test this hypothesis, we cultured BM cells isolated from β-glucan-trained mice or PBS-treated controls and performed *ex vivo* RANKL-induced osteoclastogenesis assays.^49^ BM cells isolated from trained mice were more capable of forming mature osteoclasts compared with cells isolated from untrained mice (Figures 4F and 4G). Expression of *Acp5* (encoding TRAP) in cultured osteoclasts was upregulated in cells from trained mice compared with cells from untrained mice (Figure 4H). These findings validate the hypothesis for a TRIM-triggered enhanced osteoclastogenesis bias.

**Figure 5:**
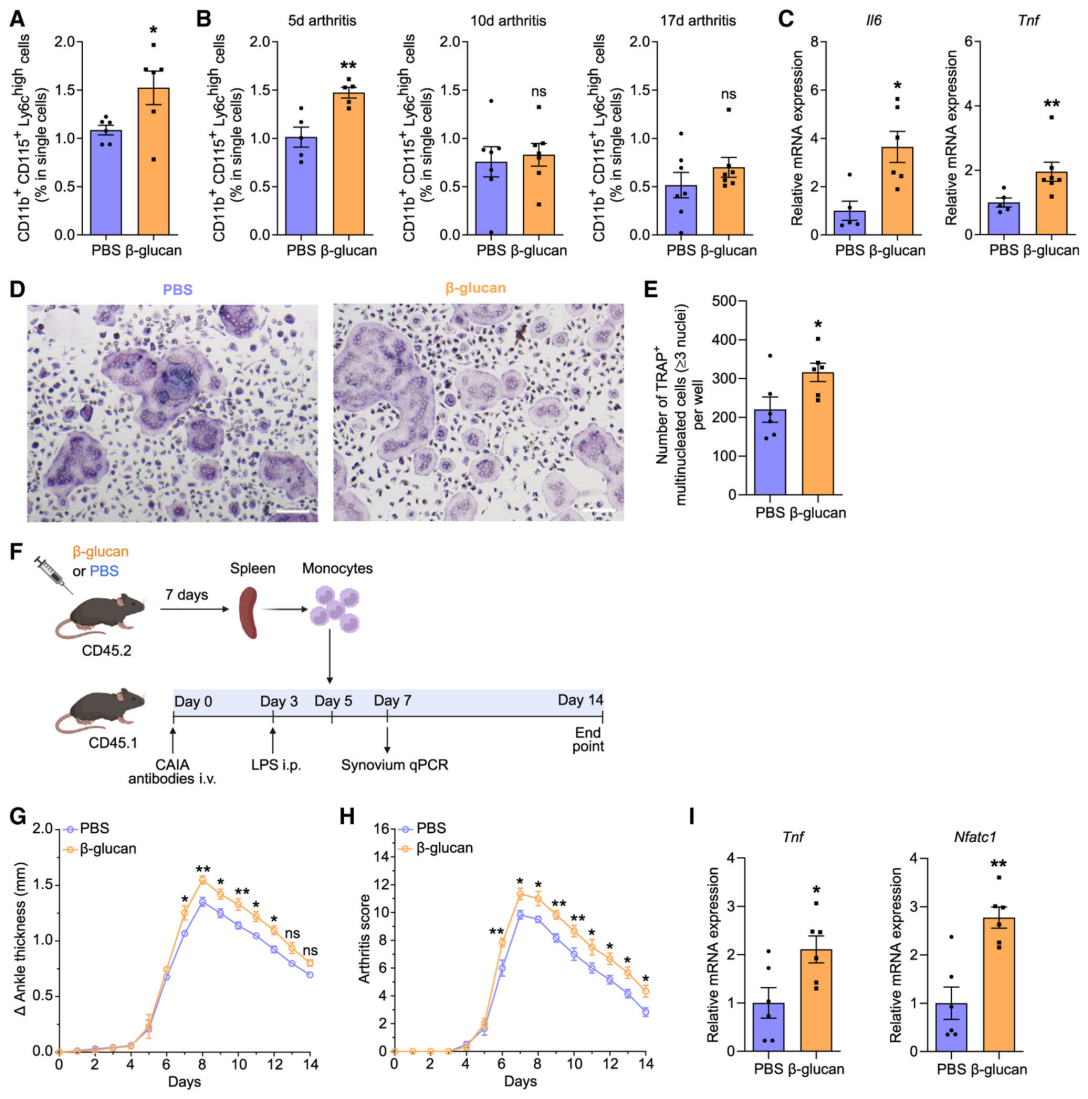
Induction of TRIM resulted in an increase in monocytes/OCPs, and adoptive transfer of trained monocytes exacerbated inflammatory arthritis (A) Mice were pre-treated with β-glucan or PBS-control and, 7 days thereafter, flow cytometry analysis for the frequency of classical monocytes (CD11b^+^ CD115^+^Ly6C^high^) in the spleen was performed (*n* = 6 mice per group). (B) Mice were pre-treated with β-glucan or PBS-control. After 7 days, both groups of mice were subjected to K/BxN-STA. Flow cytometry analysis for the frequency of classical monocytes (CD11b^+^CD115^+^Ly6C^high^) in the spleen at day 5, 10, or 17 of the K/BxN-STA model (*n* = 5–7 mice per group). (C) Mice were pre-treated with β-glucan or PBS-control. After 7 days, both groups of mice were subjected to K/BxN-STA for 17 days and classical monocytes (CD11b^+^ CD115^+^Ly6C^high^) were sorted from the spleen and processed for qPCR to measure relative mRNA expression of the indicated molecules (*n* = 5–7 mice per group). Results are presented relative to those of the PBS-control group, set as 1. (D and E) Mice were pre-treated with β-glucan or PBS-control. After 7 days, both groups of mice were subjected to K/BxN-STA for another 10 days and spleen monocytes were isolated and incubated with M-CSF and RANKL for 3 days. Cells were stained for TRAP to quantify mature osteoclasts in the culture wells, defined as TRAP+ MNCs with ≥3 nuclei. (D) Representative images (scale bars, 100 μm) and (E) quantification of osteoclasts (cultures from *n* = 6 mice per group). (F–I) CD45.2^+^ mice were pre-treated with β-glucan or PBS-control. After 7 days, spleen monocytes were isolated using the EasySep Mouse Monocyte Isolation Kit. CD45.1^+^ mice were subjected to CAIA. On day 5 of the CAIA model, monocytes from CD45.2^+^ mice were adoptively transferred by intravenous (i.v.) injection to CD45.1^+^ mice. (F) Experimental scheme. (G and H) The difference in ankle thickness, i.e., Delta (Δ) ankle thickness (G) and the arthritis score (H) are shown (*n* =6 mice per group).(I) Relative mRNA expression of the indicated molecules in knee joints, harvested on day 7 of the CAIA model (*n* = 6 mice per group). Results are presented relative to those of the PBS-control group, set as 1. Data are mean ± SEM; ns, non-significant; *p < 0.05; **p < 0.01. Unpaired t test (A, B, E, and I), except for 17 days arthritis in (B) (Mann-Whitney U test) or Mann-Whitney U test (C). Two-way ANOVA with repeated-measures and Sidak’s post-test for comparison with PBS group (G and H). See also Figures S4 and S5.

Besides the aforementioned BM OCPs that function also in homeostatic bone remodeling, under inflammatory conditions, additional myeloid cell types harbor OCP potential, including classical monocytes (CD11b^+^CD115^+^Ly6C^high^). The latter can be recruited from the blood or spleen to sites of inflammatory bone loss, where they differentiate to osteoclasts and contribute to bone resorption.^13,16^ We therefore interrogated whether TRIM can also affect monocyte-associated inflammatory osteoclastogenesis. We performed flow cytometry analysis of splenic classical monocytes (Figure S4) from β-glucan-trained mice or untrained controls for 7 days or from additional groups of trained or untrained mice that were subjected to a secondary K/BxN-STA challenge for an additional 5, 10, or 17 days. Upon β-glucan-mediated innate immune training (without further arthritis challenge), the frequency of splenic CD11b^+^CD115^+^Ly6C^high^ cells was increased compared with untrained mice (Figure 5A). Moreover, after subjecting trained and untrained mice to a secondary challenge (arthritis), CD11b^+^CD115^+^Ly6C^high^ monocytes were significantly elevated in the spleens of β-glucan-trained mice at the early phase (5 days) of the model (Figure 5B). This difference waned at later phases (10 and 17 days) of arthritis (Figure 5B). Although no differences were observed in the frequency of CD11b^+^CD115^+^Ly6C^high^ monocytes between trained and untrained mice at later stages of arthritis, splenic monocytes from previously trained mice exhibited enhanced proinflammatory signature (*Il6* and *Tnf* upregulation) 17 days after arthritis onset (Figure 5C).

Next, we assessed whether TRIM induction increased the osteoclastogenic potential of splenic monocytes, by performing *ex vivo* osteoclast differentiation assays. Splenic monocytes isolated from β-glucan-trained mice 10 days after K/BxN-STA induction exhibited enhanced osteoclast formation compared with splenic monocytes isolated from untrained arthritic mice (Figures 5D and 5E).

Hence, β-glucan-induced TRIM is associated with qualitative changes in monocytes/OCPs associated with higher inflammatory responses and enhanced osteoclastogenesis capacity. Collectively, β-glucan-induced TRIM can promote inflammatory osteoclastogenesis, at least in part, by modulating splenic monocytes/OCPs.

### Adoptive transfer of trained monocytes exacerbates inflammatory arthritis

Peripheral monocytes serve as OCPs during inflammatory osteoclastogenesis,^18^ and we found alterations in this population due to TRIM. To provide conclusive evidence that trained monocytes/OCPs promote inflammatory bone loss, we performed adoptive transfer experiments. Splenic monocytes were isolated from mice 7 days after β-glucan or PBS treatment and transferred into non-trained recipient mice that were subjected to experimental arthritis in the CAIA model 5 days prior to the adoptive transfer (Figure 5F).

The transfer of monocytes from trained mice exacerbated arthritis severity (Figures 5G and 5H). Analysis of the synovium of recipient mice 2 days after monocyte transfer revealed increased expression of the inflammatory cytokine *Tnf* and of osteoclastogenesis-promoting *Nfatc1* in mice receiving monocytes from trained donor mice compared with recipients of monocytes from untrained mice (Figure 5I). Consistent with their training toward osteoclastogenesis, monocytes isolated from β-glucan-trained mice displayed higher expression of the osteoclastogenic transcription factor *Nfatc1* than monocytes from untrained controls (Figure S5). Therefore, TRIM-induced exacerbation of inflammatory bone loss can be attributed, at least in part, to trained monocytes that are poised toward inflammatory osteoclastogenesis.

### TRIM induction is associated with transcriptomic rewiring of synovial myeloid cells

**Figure 6:**
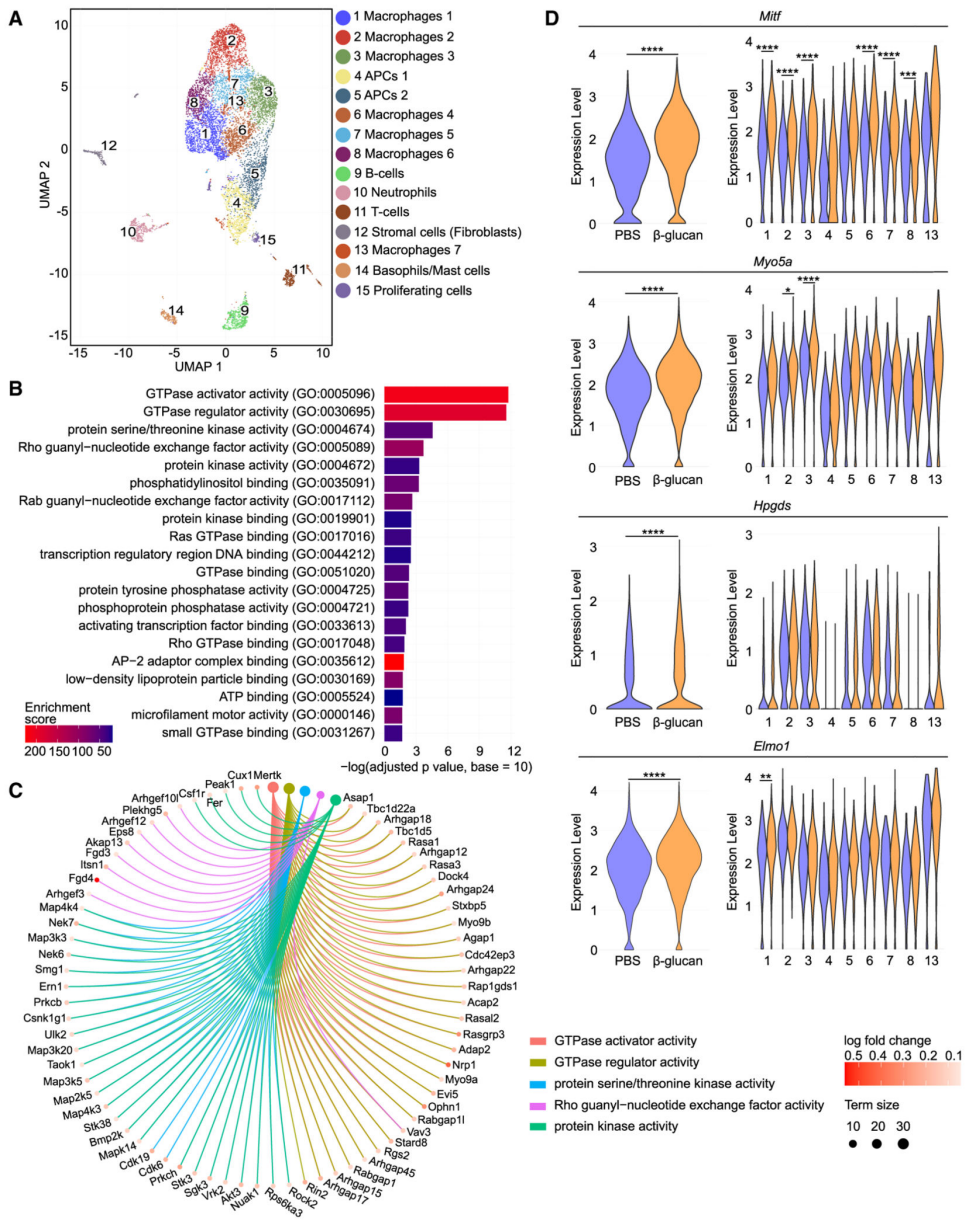
Innate immune training is associated with transcriptomic rewiring of synovial myeloid cells (A–D) Mice were pre-treated with β-glucan or PBS and 7 days later subjected to K/BxN-STA for additional 17 days and myeloid cells (CD45^+^CD11b^+^) were sorted from the hind paws and scRNA-seq analysis was performed (*n* = 4 mice per group). (A) UMAP representation from scRNA-seq of 12,956 cells. (B) Top 20 overrepresented GO terms of molecular functions of upregulated differentially expressed genes in the main myeloid cell compartment (clusters 1–8 and 13) of β-glucan-trained arthritic mice compared with PBS-control treated arthritic mice. (C) Circos plot of the top 5 overrepresented GO terms of molecular functions linked to their associated core enriched genes in the main myeloid cell compartment (clusters 1–8 and 13) of β-glucan-trained arthritic mice compared with PBS-control treated arthritic mice. “Term size” displays the number of genes annotated to the respective term. (D) Violin plots showing gene activity scores of *Mitf, Myo5a, Hpgds*, and *Elmo1* in the complete main myeloid cell compartment (left) and in all separate clusters of the main myeloid cell compartment (clusters 1–8 and 13) (right). *FDR < 0.05, **FDR < 0.01, ***FDR < 0.001, ****FDR < 0.0001. Wilcoxon rank-sum test with Bonferroni correction (D). See also Figures S6 and S7 and Tables S1 and S2.

To provide further evidence for the TRIM-inflammatory bone loss axis, we performed single-cell RNA sequencing (scRNA-seq) analysis in myeloid cells (CD45^+^CD11b^+^) from the synovium of arthritic mice that were previously trained with β-glucan or not (PBS-control). Uniform manifold approximation and projection (UMAP) of 12,956 cells (β-glucan, 7,196 cells; PBS, 5,760 cells), revealed 15 clusters (Figure 6A). The cluster identification was determined by assessing expression of cell-type-specific markers, as published,^50,51^ and by referencing the ImmGen dataset and the PanglaoDB database (Figures 6A and S6A). Annotation revealed seven macrophage clusters (clusters 1–3, 6–8, and 13) and two clusters (clusters 4 and 5) with antigen-presenting cell characteristics linked with high expression of *Cd74* and major histocompatibility complex (MHC) genes, e.g., *H2-Aa, H2-Eb1*, and *H2-Ab1*, as well as neutrophils, T cells, B cells, basophils/mast cells, proliferating cells, and stromal cells (likely representing contaminants). In subsequent analyses, we focused on the “main myeloid cell compartment,” which comprised clusters 1–8 and cluster 13 (Figures 6A and S6B).

Gene Ontology (GO) enrichment analysis of upregulated differentially expressed genes (false discovery rate [FDR] < 0.05) in the main myeloid cell compartment (clusters 1–8 and 13) between the β-glucan-trained and PBS-control groups, revealed a signature associated with small GTPase signaling in the β-glucan group (GO “molecular functions,” Figure 6B; Table S1; and GO “biological processes,” Figure S6C; Table S2). Terms such as “GTPase activator activity,” “GTPase regulator activity,” “Rho guanyl-nucleotide exchange factor activity,” and others were in the top 20 significantly enriched GO terms of upregulated differentially expressed genes (Figure 6B). Pertinently, GTPases are linked to inflammation^52,53^ and rheumatic diseases.^54^ Moreover, GTPase activity is associated with osteoclast survival, migration, and cytoskeletal remodeling.^55^ In inflammatory arthritis, the function of Rho family GTPases may underlie hyperactivation of macrophages resulting in exacerbated inflammation and bone erosion.^56,57^ Several genes related to osteoclast biology and rheumatoid arthritis were present in the top 5 upregulated GO molecular functions terms in the main myeloid cell compartment from the arthritic synovium of β-glucan-trained mice compared with untrained mice (Figure 6C). Among them, *Vav3*, encoding VAV3, and *Plekhg5*, encoding the Pleckstrin homology and RhoGEF domain containing G5 (PLEKHG5), both being guanine nucleotide exchange factors (GEFs) and activators of Rho GTPases, regulate osteoclast function.^58,59^ Additionally, *Rock2*, encoding the RhoA effector Rho-associated coiled-coil containing protein kinase 2 (ROCK2), is linked with enhanced joint inflammation in mice and increased osteoclastogenesis^60–62^ and *Ern1*, encoding IRE1α, an endoplasmic reticulum stress sensor protein, is involved in modulating arthritis-related inflammation^63,64^ and is associated with osteoclast differentiation.^65^ Moreover, *Csf1r*, encoding c-fms, also called CD115, serves as a key receptor for M-CSF, thereby playing a pivotal role in osteoclast differentiation, survival, and function.^66^ Furthermore, *Mapk14* encoding the mitogen-activated protein kinase (MAPK) p38α, a protein kinase involved in osteoclastogenesis,^67^ and its upstream activator *Map3k5*, encoding for apoptosis signal-regulating kinase 1 (ASK1), are implicated in arthritis pathogenesis.^68–70^

By further focusing on the differentially expressed genes in the clusters 1–8 and 13 of the main myeloid cell compartment from β-glucan-trained arthritic mice compared with untrained arthritic controls, we identified melanogenesis-associated transcription factor (*Mitf*) as the top ranked (according to FDR) upregulated gene when analysis was performed in the whole main myeloid cell compartment, as well as one of the top 5 genes in the individual macrophage clusters 1–3 and 6–8 (Figure 6D). *Mitf* is involved in early osteoclast development.^2,6,71^ For instance, *Mitf* is activated by IL-1 signaling and induces the expression of osteoclast-related genes in BM-derived macrophages.^21^ *Myo5a* and *Hpgds*, both described as downstream target genes of MITF and linked to GTPases,^72–74^ were also upregulated in the main myeloid cell compartment due to β-glucan pre-treatment. *Myo5a* also exhibited increased expression in clusters 2 and 3. Additionally, *Elmo1*, a gene associated with modulation of oste-oclast function and arthritis progression through GTPase activation,^75,76^ was upregulated by β-glucan-induced TRIM when analysis was performed in the whole main myeloid cell compartment, as well as in macrophage cluster 1 (Figure 6D).

*Clec5a*^*+*^ cells in the BM serve as osteoclast precursors under inflammatory conditions.^77^ Interestingly, *Clec5a* was identified as a marker gene for cluster 2 in our dataset (Figure S7A). Hence, cluster 2, which displayed transcriptomic alterations upon TRIM induction, including upregulation of *Mitf* (Figure 6D), might bear inflammatory osteoclast precursor potential. The expression of *Clec5a* itself in the main myeloid cell compartment (clusters 1–8 and 13) or in the individual clusters was not altered by β-glucan-induced TRIM (Figure S7B). These data suggest that *Clec5a*-expressing myeloid cells in the arthritic synovium are likely a target of trained osteoclastogenesis.

We also identified further osteoclast-related genes that were altered in synovial myeloid cells of arthritic mice due to β-glucan-induced TRIM. The negative regulators of osteoclastogenesis *Nr4a1* and *Klf2*^47,48,71,78^ were downregulated in the main myeloid cell compartment due to TRIM induction. Moreover, *Nr4a1* was downregulated in cluster 2, whereas *Klf2* was down-regulated in cluster 3. The transcription factor *Mef2c*, which promotes osteoclastogenesis,^79^ and *Cx3cr1*, which is expressed by osteoclast precursors and involved in their recruitment,^12,14^ were upregulated in the main myeloid cell compartment of β-glucan-trained arthritic mice compared with untrained arthritic controls (Figure S7C).

Together, TRIM mediates transcriptomic rewiring of myeloid cells/macrophages in the arthritic synovium, encompassing enrichment of a GTPase-signaling signature and featuring prominent upregulation of *Mitf* and of further genes linked to inflammation and regulation of osteoclast development and function. These findings are consistent with innate immune training of inflammatory osteoclastogenesis and the associated disease phenotypes described earlier in the present study.

### MITF mediates the arthritis-promoting function of trained monocytes

**Figure 7:**
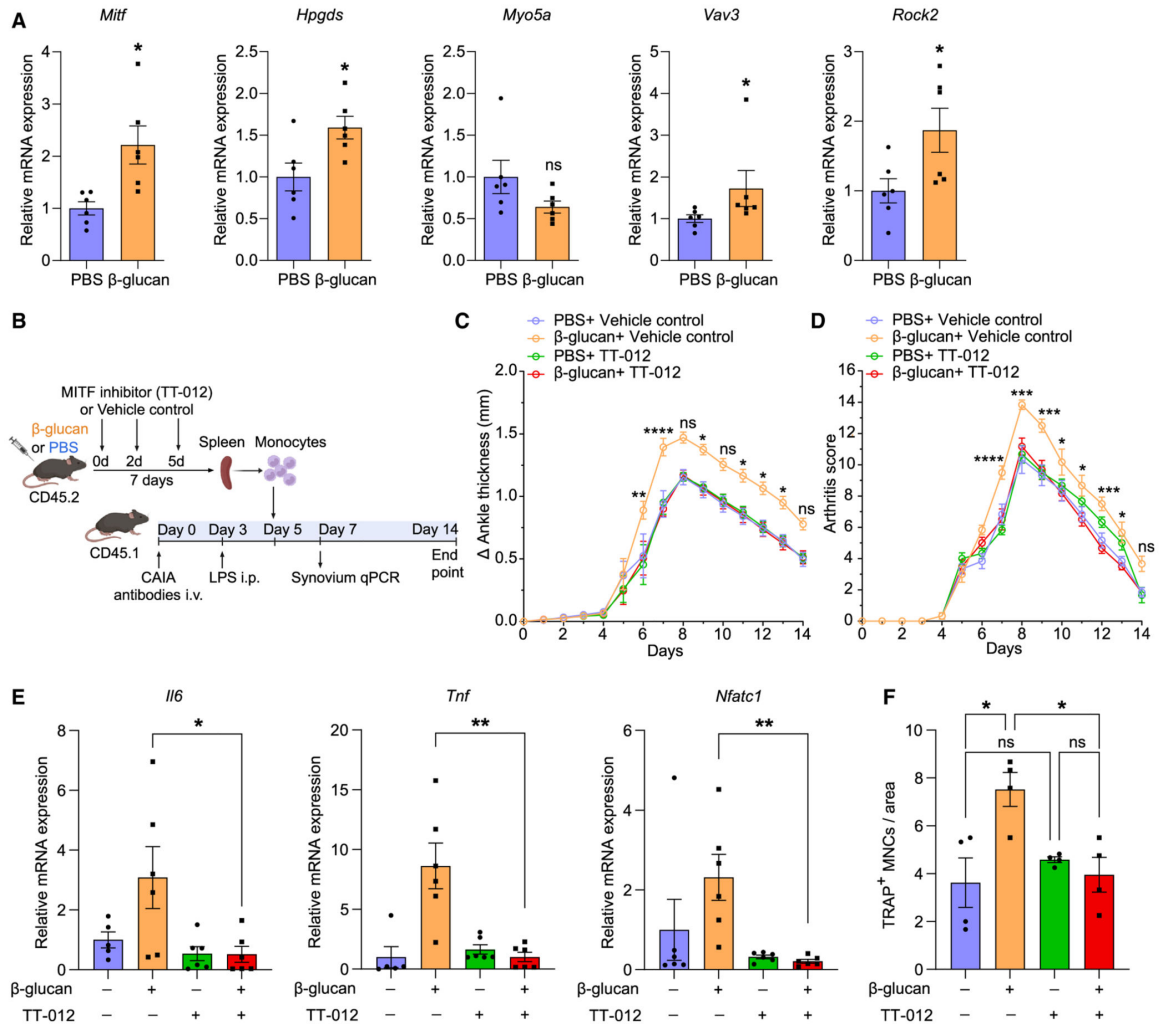
MITF mediates the arthritis-promoting function of trained monocytes (A) Mice were treated with β-glucan or PBS-control and, after 7 days, splenic monocytes were isolated using the EasySep Mouse Monocyte Isolation Kit. Relative mRNA expression of the indicated molecules; results are presented as fold change relative to the PBS group, set as 1 (*n* = 6 mice per group). (B–F) CD45.2^+^ mice were pre-treated with β-glucan or PBS-control and additionally received the MITF inhibitor TT-012 or vehicle control on days 0, 2, and 5. After 7 days, spleen monocytes were isolated using the EasySep Mouse Monocyte Isolation Kit. CD45.1^+^ mice were subjected to CAIA. On day 5 of the CAIA model, monocytes from CD45.2^+^ mice were adoptively transferred by i.v. injection to CD45.1^+^ mice. (B) Experimental scheme. (C and D) The difference in ankle thickness, i.e., Delta (Δ) ankle thickness (C) and the arthritis score (D) are shown (*n* = 6 mice per group). Significant differences are shown between β-glucan + vehicle control vs. β-glucan + TT-012 at the respective days. (E) Relative mRNA expression of the indicated molecules in knee joints, harvested on day 7 of the CAIA model (*n* = 5–6 mice per group). Results are presented as fold change in the transcript levels relative to those of PBS + vehicle control (assigned an average value of 1). (F) TRAP staining of knee joints (harvested on day 7 of the CAIA model) to quantify TRAP^+^ MNCs per area (*n* = 4 mice per group). Data are mean ± SEM; ns, non-significant; *p < 0.05; **p < 0.01; ***p < 0.001; ****p < 0.0001. Unpaired t test (A and E) except for *Myo5a* and *Vav3* in (A) (Mann-Whitney U test); one-way ANOVA with Tukey’s multiple comparison test (F). Two-way ANOVA with repeated-measures and Sidak’s post-test for comparison between β-glucan + vehicle control vs. β-glucan + TT-012 (C and D).

We next assessed whether the TRIM-mediated transcriptomic rewiring of macrophages, as identified in the arthritic synovium (Figure 6), is already initiated in their precursors, in particular, in splenic monocytes/OCPs, thereby providing additional evidence for the process of trained osteoclastogenesis. We isolated splenic monocytes from β-glucan-trained and untrained mice (without further arthritis induction) and studied the expression of factors linked to the TRIM-mediated transcriptomic rewiring of synovial macrophages of arthritic mice. Strikingly, the expression of *Mitf* and of the MITF downstream target *Hpgds* (but not of *Myo5a*) was significantly enhanced in splenic monocytes from β-glucan-trained compared with cells from control-treated mice (Figure 7A). Moreover, mRNA expression of VAV3 RhoGEF and the RhoA effector ROCK2 was elevated in splenic monocytes from β-glucan-trained compared with untrained mice (Figure 7A). Thus, the TRIM-induced transcriptomic rewiring occurs as early as at the level of splenic monocytes/OCPs.

As *Mitf* upregulation was the most prominent feature of TRIM-induced transcriptomic rewiring in synovial macrophages in arthritis, and already detectable in splenic monocytes/OCPs from trained mice, we next assessed the function of MITF in mediating innate immune training of inflammatory osteoclasto-genesis and exacerbation of inflammatory bone loss. Splenic monocytes were isolated from mice that were treated 7 days before with β-glucan or PBS-control, followed by additional treatment with the MITF inhibitor TT-012 or vehicle control, and subsequently transferred into naive recipient mice that were subjected to experimental arthritis in the CAIA model 5 days prior to the adoptive transfer (Figure 7B). As compared with control treatment, MITF inhibition in donor mice blocked the ability of monocytes from β-glucan-trained donor mice to exacerbate arthritis severity in recipient mice (Figures 7C and 7D), as well as to increase expression of the inflammatory cytokines *Il6* and *Tnf* and of the osteoclastogenic transcription factor *Nfatc1* in the synovium of recipient arthritic mice (Figure 7E). Furthermore, the transfer of monocytes from β-glucan-trained donor mice to arthritic mice resulted in an increase in osteoclasts in the arthritic joints, compared with monocyte transfer from non-trained mice (Figure 7F). Administration of the MITF inhibitor (compared with vehicle control) in donor mice abrogated the effect of the transferred trained monocytes to enhance osteoclast numbers in the knee joints of recipient arthritic mice (Figure 7F). In contrast, MITF inhibition in donor mice did not alter osteoclast numbers in arthritic mice receiving non-trained monocytes from PBS-control-treated mice (Figure 7F). These data indicate that adoptive transfer of monocytes from trained mice increases osteoclastogenesis in recipient arthritic mice in a manner that requires MITF signaling during the process of TRIM induction in donor mice.

Collectively, these data establish the transcription factor MITF as critical for the process by which TRIM promotes inflammatory osteoclastogenesis at the level of monocytes and thereby exacerbates inflammatory bone loss.

## Discussion

TRIM has emerged as a major immunological principle that challenged the dogma that memory is restricted to adaptive immunity.

We and others have recently established that the long-term actions of TRIM are linked to rewiring of BM hematopoietic progenitors.^24,26,27,29^ In this context, β-glucan-induced innate immune training of specific arms of myelopoiesis, e.g., granulopoiesis, leads to potent anti-tumor activity.^30^ Here, we show that an additional myelopoiesis arm, namely the generation of myeloid precursors of osteoclasts and osteoclastogenesis, is also targeted by TRIM. In this setting, however, TRIM has detrimental consequences, contributing to inflammatory bone loss. TRIM has therefore context-dependent, beneficial or detrimental, effects. Understanding this duality is imperative to appropriately harness TRIM for therapeutic gain in human disease.

Administration of β-glucan promoted OCP expansion and qualitatively modulated different OCP populations, enhanced osteoclastogenesis in the BM and inflammatory osteoclastogenesis in the periphery, and, thereby, aggravated inflammatory bone loss pathologies. Importantly, β-glucan pre-treatment induced transcriptomic changes indicative of a bias toward increased osteoclast differentiation, especially in CD115^+^CD27^low/−^ OCPs. These results align with prior findings demonstrating transcriptional rewiring in BM myeloid progenitors upon TRIM induction.^27–30,40^ Thus, β-glucan-induced TRIM not only targets myelopoiesis/granulopoiesis at the level of GMPs^30^ but also promotes an osteoclastogenesis bias in progenitors downstream of GMPs. Furthermore, we have recently demonstrated that maladaptive TRIM induced by chronic experimental periodontitis is associated with epigenetic and transcriptomic changes in HSPCs involving an over-representation of genes linked to “osteoclast differentiation” pathways.^40^ In other words, notwithstanding its aforementioned protective effects against infection or cancer,^24–26,30^ β-glucan-induced trained myelopoiesis can be maladaptive by potentiating osteoclastogenesis and hence inflammatory bone loss in the context of chronic inflammation.^40^ Our findings with β-glucan in this and our earlier work^30^ also argue against the notion that beneficial and maladaptive forms of TRIM are driven by different stimuli; the findings rather suggest that the context in which TRIM emerges dictates whether the functional outcome is protective or inappropriate to the specific situation. Monocytes are major cellular effectors of β-glucan-induced TRIM, and trained monocytes display heightened production of inflammatory cytokines in response to secondary challenges.^24,32–34,40^ Consistently, we found that β-glucan-mediated TRIM was associated with the upregulation of proinflammatory cytokines and transcription factors that drive inflammatory osteoclastogenesis.^2,18,80^ Moreover, β-glucan-induced TRIM increased the abundance of splenic CD11b^+^CD115^+^Ly6C^high^ monocytes, which have OCP potential,^13^ and enhanced their inflammatory capacity upon a secondary challenge, specifically arthritis. Such monocytes/OCPs expand in the periphery in the setting of inflammatory bone loss disorders and migrate to the inflamed site, where they can differentiate into osteoclasts.^13,16^ Consistently, in *ex vivo* osteoclast differentiation assays, β-glucan-induced TRIM enhanced the inflammatory osteoclastogenic potential of splenic monocytes/OCPs in arthritic mice. As osteoclasts can derive from a broad range of myeloid cell populations bearing OCP potential,^18^ our results suggest that innate immune training of osteoclastogenesis by β-glucan can occur at different levels, rewiring both BM and peripheral OCP populations. Although there was a predisposition toward enhanced osteoclastogenesis in BM and peripheral OCP populations in mice due to TRIM induction, the number of osteoclasts in the joints and/or the osteoclastic activity increased only upon the arthritis challenge as a secondary stimulus. These findings epitomize induction of TRIM (higher responsiveness upon a secondary stimulus) in the osteoimmunological context and unequivocally support the concept of trained osteoclastogenesis.

Monocyte-mediated inflammatory osteoclastogenesis was an integral component of the effect of β-glucan-induced TRIM to exacerbate inflammatory bone loss. In this regard, we identified a transcriptomic rewiring in synovial macrophages of β-glucan-trained arthritic mice. Pathways related to activity of GTPases, which orchestrate myeloid cell recruitment and activation, macrophage activation, osteoclastogenesis, and inflammatory bone erosion in inflammatory arthritis,^54–57,81^ were overrepresented in synovial myeloid cells/macrophages from β-glucan-trained arthritic mice. Furthermore, the top ranked upregulated gene in synovial myeloid cells/macrophages from trained arthritic mice, *Mitf*, encoding for a transcription factor involved in osteoclastogenesis,^6,71^ has also been implicated in activating GTPase signaling.^82^ The MITF target genes *Myo5a* and *Hpgds*, which were also upregulated in synovial myeloid cells/macrophages due to β-glucan-mediated TRIM, are similarly linked to GTPase signaling.^72–74^ Notably, the TRIM-induced transcriptomic rewiring was evident as early as the level of splenic monocytes/OCPs because expression of *Mitf, Hpgds, Rock2*, and *Vav3* was also upregulated in monocytes from β-glucan-trained mice. Adoptive transfer of monocytes from β-glucan-trained donor mice was sufficient to exacerbate inflammatory bone loss in recipient arthritic mice, accompanied by elevated osteoclast numbers in the arthritic joints, as compared with arthritic mice that received untrained monocytes; this effect was critically mediated by the transcription factor MITF. As MITF is an upstream activator of GTPase signaling in non-hematopoietic cells,^82^ our findings enable us to hypothesize that MITF orchestrates trained inflammatory osteoclastogenesis in monocytes and drives their transcriptomic rewiring toward enhanced GTPase activity, which, in turn, further contributes to aggravated inflammatory bone loss.^54–57,81^ Together, β-glucan-induced TRIM promotes MITF-dependent monocyte-mediated inflammatory osteoclastogenesis, thereby exacerbating inflammatory bone loss.

The double-edged sword nature of TRIM, with its detrimental facets underlying the pathogenesis of chronic inflammation and autoimmune disease,^28,40,83–85^ acquires special relevance when considering the preventive or therapeutic application of TRIM-inducing agents.^24^ Pertinently, β-glucan is currently tested in clinical trials as an adjuvant in cancer immunotherapy, mostly in combination with immune checkpoint inhibitors.^41,42,86,87^ Immune checkpoint inhibitors are linked to immune-mediated adverse events, including conditions such as rheumatoid arthritis-like joint inflammation and bone loss.^43,44,88^ Our findings therefore suggest that the use of β-glucan as an adjuvant in immunotherapies, especially in combination with immune check-point inhibitors, might potentially worsen these side effects or unmask autoimmunity during treatment. Additional investigations are warranted toward better understanding and preventing such potential side effects linked to the therapeutic use of β-glucan in light of the beneficial actions of the molecule in cancer. Moreover, the capacity of certain adjuvants to trigger autoimmunity or autoinflammation has resulted in the emergence of the autoimmune/autoinflammatory syndrome induced by adjuvants (ASIA).^89^ Different β-glucans have been used in the past as adjuvants in distinct experimental models of autoimmunity.^90–97^ Although these earlier studies indicate a link between β-glucan administration and increased inflammation, they did not implicate β-glucan-induced TRIM in this context. Our present study clearly suggests that β-glucan-induced TRIM might also bear the risk for triggering an ASIA syndrome. Importantly, in the context of β-glucan therapies, pharmacological MITF inhibition could prevent trained inflammatory osteoclastogenesis without in principle interfering with the intended beneficial effects (e.g., actions against infections or cancer). In conclusion, our work underscores that detailed understanding of the beneficial and detrimental actions of β-glucan-induced TRIM and the underlying molecular mechanisms is imperative for developing effective therapies that leverage this principle.

## Limitations of the study

Our work introduces the concept of innate immune training of (inflammatory) osteoclastogenesis, which may promote inflammatory bone loss. Specifically, we found that β-glucan administration modulated OCP populations in the BM and the spleen. It is conceivable that the enhanced osteoclastogenesis bias upon TRIM induction is initiated at the level of earlier BM progenitors, namely at HSPCs. However, this possibility was not addressed here and merits future investigations. Additionally, our work identified that the transcription factor MITF contributes to trained inflammatory osteoclastogenesis in monocytes. Our conclusion is based on studies engaging pharmacological MITF inhibition; this approach bears some limitations, as MITF could be targeted not only in monocytes but also in additional cells. Future studies should therefore assess the role of MITF in β-glucan-induced TRIM and inflammatory osteoclastogenesis by using genetic tools: for instance, mice with MITF inactivation specifically in myeloid cells.

## Resource Availability

## Lead contact

Requests for further information and resources should be directed to and will be fulfilled by the lead contact, Triantafyllos Chavakis (triantafyllos.chavakis@ ukdd.de).

## Materials availability

This study did not generate new unique reagents.

## Data and code availability

- Data are available upon request to the lead contact.
- Sequencing data are available at the Gene Expression Omnibus (https://www.ncbi.nlm.nih.gov/geo/) under accession number GEO: GSE254560.
- Any additional information required to reanalyze the data reported in this work paper is available from the lead contact upon request.

## Star+Methods

Detailed methods are provided in the online version of this paper and include the following:

- Key resources table
- Experimental model and study participant details
  - Mice
- Method details
  - Induction and evaluation of LIP
  - Induction and evaluation of experimental arthritis
  - Adoptive Transfer Model
  - Tissue preparation
  - Flow cytometry and sorting
  - Ex vivo osteoclast differentiation and Leukocyte Acid Phosphatase (TRAP) staining
  - Histology
  - Serum analysis
  - RNA isolation and quantitative PCR analyses
  - Single-cell RNA sequencing
- Quantification and statistical analysis
  - Single-cell RNA sequencing
  - Statistical analysis

## Star+Methods

### Key Resources Table

**Table:** 

REAGENT or RESOURCE	SOURCE	IDENTIFIER
Antibodies		
Armenian Hamster anti-mouse CD27	Thermo Fisher Scientific	Cat# 17-0271-82; RRID: AB_469370
Armenian Hamster anti-mouse CD27	BioLegend	Cat# 124208; RRID: AB_1236466
Rat anti-mouse CD115	Thermo Fisher Scientific	Cat# 12-1152-82; RRID: AB_465808
Rat anti-mouse CD115	BioLegend	Cat# 135524; RRID: AB_2566460
Rat anti-mouse CD117 (c-Kit)	Thermo Fisher Scientific	Cat# 47-1171-82; RRID: AB_1272177
Rat anti-mouse CD117 (c-kit)	BioLegend	Cat# 105808; RRID: AB_313217
Rat anti-mouse CD11b	Thermo Fisher Scientific	Cat# 45-0112-82; RRID: AB_953558
Rat anti-mouse CD11b	Thermo Fisher Scientific	Cat# 25-0112-81; RRID: AB_469587
Rat anti-mouse CD11b	BioLegend	Cat# 101216; RRID: AB_312799
Rat anti-mouse CD11b	BioLegend	Cat# 101212; RRID: AB_312795
Rat anti-mouse/human CD11b	BioLegend	Cat# 101208; RRID: AB_312791
Rat anti-mouse CD45R (B220)	Thermo Fisher Scientific	Cat# 56-0452-82; RRID: AB_891458
Rat anti-mouse CD45	BioLegend	Cat# 103112; RRID: AB_312977
Rat anti-mouse CD45	BioLegend	Cat# 103106; RRID: AB_312971
Rat anti-mouse CD45R (B220)	BioLegend	Cat# 103232; RRID: AB_493717
Rat anti-mouse F4/80	Thermo Fisher Scientific	Cat# 25-4801-82; RRID: AB_469653
Rat anti-mouse Ly-6C	BioLegend	Cat# 128032; RRID: AB_2562178
Rat anti-mouse Ly-6G	BioLegend	Cat# 127606; RRID: AB_1236494
Biological Samples		
K/BxN serum from arthritic mice	generated from K/BxN mice, as previously (Sormendi et al.^98^)	N/A
Chemicals, peptides, and recombinant proteins		
Beta-glucan peptide (BGP)	Invivogen	Cat# tlrl-bgp
Glucan from baker’s yeast(*S. cerevisiae)*	Sigma-Aldrich	Cat# G5011
TT-012 (MITF Inhibitor)	MedChemExpress	Cat# HY-156483
Acetone	Sigma-Aldrich	Cat# 1.00014
Bovine serum albumin (BSA), FA free	Sigma-Aldrich	Cat# A7030
Chloroform	Carl Roth	Cat# 4432.1
Collagenase from *Clostridium histolyticum*	Sigma-Aldrich	Cat#C5138
Collagenase type 4 from *Clostridium histolyticum*	Worthington	Cat# LS004186
Corn oil	MedChemExpress	Cat# HY-Y1888
DMSO	MedChemExpress	Cat# HY-Y0320
DNase I	Roche	Cat# 10104159001
DNase I recombinant	Roche	Cat# 04536282001
Ethanol absolute	VWR Chemicals	Cat# 20821.310
Fetal Bovine Serum (FBS)	Gibco	Cat# 10270-106
Formaldehyde solution	Sigma-Aldrich	Cat# 252549
GlutaMAX supplement	Gibco	Cat# 35050061
MEM α, nucleosides	Thermo Fisher Scientific	Cat# 22571038
PBS (10X), pH 7.4	Gibco	Cat# 70011051
Penicillin-Streptomycin	Gibco	Cat# 15140122
RBC Lysis Buffer (10X)	eBioscience	Cat# 00-4300-54
Recombinant Mouse M-CSF Protein	R&D systems	Cat# 416-ML-010/CF
Recombinant Mouse TRANCE/RANK L/TNFSF11	R&D systems	Cat# 462-TEC-010/CF
RPMI 1640 Medium	Gibco	Cat# 21875091
TRI Reagent	MRC	Cat#TR118
TRIzol™ Reagent	Invitrogen	Cat# 15596026
UltraPure™ 0.5M EDTA, pH 8.0	Invitrogen	Cat# 15575020
DAPI (4’,6-Diamidin-2-phenylindol, Dihydrochlorid)	Invitrogen	Cat# D1306
Hoechst 33258, Pentahydrate (bis-Benzimide)	Invitrogen	Cat# H1398
Trypan Blue Solution, 0.4%	Gibco	Cat# 15250061
Sucrose	Sigma-Aldrich	Cat# S9378-1KG
Tissue-Tek™ O.C.T. Compound	Sakura Finetek	Cat# 4583
O.C.T. Compound Cryostat Embedding Medium	Scigen	Cat# 23-730-625
Formic acid	Sigma-Aldrich	Cat# F0507-500ML
Immunocal Decalcifier	StatLab	Cat#STL14141
Formalin 37%	Morphisto	Cat# 15071.00250
Acetone	Merck	Cat# 179124-500ML
Formalhehyde solution 4%	SAV	Cat# FN-1000-4-1
Critical commercial assays		
Arthrogen-CIA® 5-Clone Cocktail Kit	Chondrex	Cat# 53010
Chromium Next GEM Chip G Single Cell Kit (48 rxns)	10x Genomics	Cat# PN-1000120
Chromium Next GEM Single Cell 3’ Kit v3.1 (16 rxns)	10x Genomics	Cat# PN-1000268
Dual Index Kit TT Set A 96 rxns	10x Genomics	Cat# PN-1000215
EasySep™ Mouse Monocyte Isolation Kit	STEMCELL	Cat# 19861
EasySep™ PE Positive Selection Kit II	STEMCELL	Cat# 17684
TaqMan™ Fast Advanced Master Mix for qPCR	Applied Biosystems	Cat# 4444557
GeneJET RNA Purification Kit	Thermo Fisher Scientific	Cat# K0731
High-Capacity cDNA Reverse Transcription Kit	Applied Biosystems	Cat# 43-688-14
iScript cDNA Synthesis Kit	BioRad	Cat# 1708891
Leukocyte Acid Phosphatase (TRAP) Kit	Sigma	Cat# 387A-1 KT
TRAP Staining Kit	Cosmo Bio LTD	Cat# PMC-AK04F
NovaSeq 6000 S4 Reagent Kit v1.5 (200 cycles)	Illumina	Cat# 20028313
NovaSeq 6000 Xp 4-lane Kit v1.5	Illumina	Cat# 20043131
RNeasy Plus Micro Kit	QIAGEN	Cat# 74034
SPRIselect beads (450 ml)	Beckman Coulter	Cat# B23319
SsoFast EvaGreen Supermix	BioRad	Cat# 1725204
MouseTRAP™ (TRAcP 5b) ELISA	IDS	Cat# SB-TR103
Deposited data		
Single cell RNA sequencing data	This paper	GEO: GSE254560
Experimental models: Organisms/strains		
Mouse: C57BL/6	The Jackson Laboratory	Stock #000664
Mouse: C57BL/6	Janvier Labs	C57BL/6JRj
Mouse: C57BL/6	Charles River	C57BL/6JCrl
Mouse: C57BL/6-Tg(UBC-GFP)30Scha/J	The Jackson Laboratory	Stock #004353
Mouse: C57BL/6.SJL CD45.1 +	The Jackson Laboratory	Stock #002014
Software and algorithms		
BioRender	BioRender	https://www.biorender.com/
FlowJo version 10	Tree Star	https://www.flowjo.com/solutions/flowjo
GraphPad Prism 10.1.2	Graphpad Software	https://www.graphpad.com/
Cell Ranger software (v7.0.0)	10X Genomics	https://support.10xgenomics.com/single-cell-gene-expression/software/release-notes/build#mm10_2020A
R Version 4.2.2	R Foundation	https://www.r-project.org/
Other		
ImmGen dataset	Immunological Genome Project	https://www.immgen.org/
PanglaoDB	Franzen et al.^99^	https://panglaodb.se/index.html

### Experimental Model and Study Participant Details

#### Mice

Wild-type (WT) C57BL/6 mice were purchased from Charles River, Janvier or Jackson Laboratory. UBC-GFP H-2^d^ mice were obtained from the Jackson Laboratory (strain # 004353). Mice were kept under specific pathogen-free conditions on a standard 12-h light/dark cycle. Food and water were provided *ad libitum*. The mice were 7-10 weeks old at the start of the experiments. Animal experiments were approved by the Landesdirektion Sachsen, Germany, the Institutional Animal Care and Use Committee of the University of Pennsylvania and the Research Ethics Committee of West China Hospital of Stomatology. To investigate the role of TRIM on inflammatory bone loss diseases, mice were pre-treated with a single i.p injection of 1 mg β-glucan peptide from *Trametes versicolor* (Invivogen), or 1 mg β-glucan from *S. cerevisiae* (Sigma-Aldrich), both dissolved in PBS, or PBS alone (PBS-control). After 7 days, mice were either euthanized or subjected to a secondary inflammatory challenge by inducing experimental periodontitis or experimental arthritis, as described in the respective paragraphs.

## Method Details

### Induction and evaluation of LIP

Ligature-induced periodontitis (LIP) generates a localized environment that retains biofilm, resulting in inflammation and subsequent bone loss.^40,100–103^ LIP was performed in mice as previously described.^100^ Briefly, a 5-0 silk ligature was tied around the maxillary left second molar tooth, whereas the contralateral molar tooth was kept unligated to serve as baseline control. The mice were euthanized 5 days later. Defleshed maxillae were used to measure bone heights (i.e., the distances from the cementoenamel junction [CEJ] to the alveolar bone crest [ABC]) at six predetermined points on the ligated site as previously specified.^100^ Measurements were made using a dissecting microscope fitted with a video image marker measurement system (Nikon Instruments). To calculate bone loss, the six-site total CEJ-ABC distance for the ligated site of each mouse was subtracted from the six-site total CEJ-ABC distance of the contralateral unligated site. The results are presented in millimeters, and negative values indicate bone loss relative to the baseline (unligated control).

### Induction and evaluation of experimental arthritis

CAIA was performed in mice as previously described.^40,104,105^ Briefly, CAIA was induced by intravenous (i.v.) injection of 1.5 mg arthritogenic mAbs (Arthrogen-CIA 5-Clone Cocktail Kit; Chondrex) per mouse on day 0, followed by i.p. injection of 50 μg lipopolysaccharide (LPS; Chondrex) on day 3. Hind ankle joint thickness was recorded and clinical arthritis scores were evaluated according to a previously described scoring system on a 0 to 4 scale^104^: 0 for normal; 1 for mild redness, slight swelling of ankle or wrist; 2 for moderate swelling of ankle or wrist; 3 for severe swelling, including some digits, ankle and foot; 4 for maximally inflamed joint. The final clinical score for each mouse was the sum of the scores in all 4 paws (maximum 16 points per mouse).

In other experiments, K/BxN serum transfer arthritis (STA) was induced in mice by i.p. injection of 150 μL K/BxN serum on day 0 and 2.^98^ The progression of arthritis was monitored by recording ankle joint thickness and clinical arthritis scores. Ankle joint thickness was measured using a digital caliper, and the average thickness of both hind paws was calculated for each mouse. Arthritis scores were evaluated based on a previously described scoring system.^106^ In detail, hindlegs and forelegs were scored on a scale ranging from 0 to 3 points whereas the points were given as follows: 0, no swelling and no redness; 1 for mild redness or slight swelling of ankle or wrist; 2 moderate swelling of ankle or wrist; 3 for severe swelling, including digits, ankle, and foot. Arthritis score and ankle thickness was evaluated in a blinded manner. The final score for each mouse was determined as the sum of scores for all 4 paws (maximum 12 points per mouse). The difference in ankle thickness (“delta ankle thickness”) for each mouse was calculated by subtracting the average ankle thickness on the day of arthritis initiation (day 0) from the average ankle thickness at each time point throughout the experiment.

### Adoptive Transfer Model

Donor mice (CD45.2^+^ UBC-GFP) were pre-treated with β-glucan (1 mg/mouse) or PBS as a control. After 7 days, splenic monocytes were sorted using the EasySep Mouse Monocyte Isolation Kit (Stemcell). Recipient C57BL/6.SJL CD45.1^+^ mice (B6.SJL-*Ptprc*^*a*^*Pepc*^*b*^/BoyJ) were subjected to CAIA, and 5 days later, 1x10^6^ sorted monocytes from CD45.2^+^ UBC-GFP donor mice were adoptively transferred via i.v. injection to CD45.1^+^ recipient mice; arthritis development was evaluated as described under “Induction and evaluation of experimental arthritis”.

In other experiments, donor mice (C57BL/6 from Jackson Laboratory) were pre-treated with β-glucan (1 mg/mouse) or PBS as a control, and received also the MITF inhibitor TT-012 i.p. (20 mg/kg; MedChemExpress), dissolved in 10% DMSO and 90% corn oil, or treated with the vehicle control on days 0, 2 and 5 after β-glucan or PBS injection. After 7 days, splenic monocytes were isolated using the EasySep™ Mouse Monocyte Isolation Kit (Stemcell) and transferred to recipient mice. Specifically, C57BL/6.SJL CD45.1^+^ recipient mice (B6.SJL-*Ptprc*^*a*^*Pepc*^*b*^/BoyJ) were subjected to CAIA and 5 days later received 1x10^6^ monocytes from the aforementioned donor groups via i.v. injection. Arthritis development was evaluated as described under ‘Induction and evaluation of experimental arthritis’.

### Tissue preparation

Synovial tissue from ankle joints underwent processing as previously described.^51^ Specifically, ankle joints were isolated, and cells were dissociated at 37°C and 90 r.p.m. for 45 minutes in a digestion medium containing RPMI culture medium, 10% fetal bovine serum (FBS), 2 mg/ml collagenase from *Clostridium histolyticum* (Sigma-Aldrich), and 0.03 mg/ml DNase I (Roche). Cells were passed through a 40 μm filter, and erythrocytes were lysed using red blood cell (RBC) lysis buffer (eBioscience). Single cell suspensions were washed and resuspended for flow cytometry analysis or cell sorting. In other experiments, synovial tissue from the knee joint was extracted and digested for 30 min at 37°C in DMEM containing 10% FBS, penicillin/streptomycin, 2 mg/mL collagenase type IV (Worthington) and 0.1 mg/mL DNase I (Roche). Synovial myeloid cells were then isolated by positive selection for CD11b^+^ cells (clone M1/70; BioLegend), followed by isolation of CD45^-^ cells to obtain synovial stromal cells (clone 30-F11; BioLegend) using the EasySep™ PE Positive Selection Kit II (Stemcell), according to the manufacturer’s instructions.

Mouse spleens were homogenized, and BM was crushed using ice-cold PBS with 5% FBS. Upon erythrocyte lysis with RBC lysis buffer (eBioscience), cells were centrifuged at 500 x g for 5 minutes. Cells were forced through a 40 μm cell filter to get single–cell suspension for further flow cytometric analysis or FACS cell sorting.

### Flow cytometry and sorting

Flow cytometry was performed by FACS Fortessa (BD, Germany) and cell sorting was performed on FACS Aria cell sorters (BD, Germany), or a FACS Aria II (BD, USA). For cell surface phenotypic analysis, isolated murine cells were stained with the following monoclonal antibodies: Anti-CD117/c-Kit (clone 2B8), anti-CD45 (clone 30-F11), anti-CD11b (clone M1/70), anti-Ly6G (clone 1A8), anti-Ly6C (clone HK1.4), anti-F4/80 (clone BM8), anti-CD115 (clone AFS98), anti-B220 (clone RA3-6B2), anti-CD27 (clone LG.7F9 or LG.3A10). Stained cells were then subjected to flow cytometry or cell sorting. Data analysis was carried out using FlowJo software (Tree Star).

### Ex vivo osteoclast differentiation and Leukocyte Acid Phosphatase (TRAP) staining

Femoral, tibial and humeral bones from C57BL/6 mice were flushed under sterile conditions using cell culture medium. Following erythrocyte lysis with RBC lysis buffer (eBioscience), BM cells were cultured in 48 well plates (for TRAP staining) or in 12 well plates (for qPCR analysis) with 25 ng/mL M-CSF (R&D Systems) in α-MEM supplemented with 10% FBS, 1% penicillin-streptomycin, and 1% GlutaMAX. After 3 days, 50 ng/mL RANKL (R&D Systems) was added, and cells were further cultured for 5 days to induce osteoclast differentiation.

In other experiments, splenic monocytes were isolated using the EasySep™ Mouse Monocyte Isolation Kit (Stemcell) according to the manufacturer’s instructions. Cells were cultured at a density of 50,000 cells per well in a 96-well plate for 3 days in a culture medium containing 25 ng/mL M-CSF (R&D Systems), 50 ng/mL RANKL (R&D Systems) and α-MEM supplemented with 10% FBS, 1% penicillin-streptomycin and 1% GlutaMAX.

Osteoclast development was evaluated by counting all TRAP-positive cells with 3 or more nuclei in each well under a microscope with a 20x objective. For TRAP staining, *in vitro* cultured BM- or spleen-derived osteoclasts were fixed for 10 minutes in a fixation solution containing citrate solution, acetone and 37% formaldehyde. Staining for TRAP was performed by using a leukocyte acid phosphatase kit (Sigma-Aldrich), following the manufacturer’s recommendations with minor modifications. TRAP-positive multinu-cleated cells ([MNCs]; ≥ 3 nuclei) were defined as bona fide osteoclasts.

### Histology

Knee joints were harvested and fixed in 4% paraformaldehyde. The tissues were then decalcified in 5% formic acid for 2 to 3 weeks, followed by immersion in either 10%, 20%, 30%, or only in 30% sucrose in PBS. Samples were embedded in optimal cutting temperature compound and sections were cut at 7-10 μm. TRAP staining was performed using a leukocyte acid phosphatase kit (Sigma-Aldrich) or a TRAP Staining Kit (Cosmo Bio). TRAP^+^ MNCs with 3 or more nuclei were considered as osteoclasts. Osteoclast quantification was performed by counting all TRAP^+^ MNCs in 4 distinct areas and 3 sections within the knee joint either under a microscope at 400x magnification or from captured images. The average of all counted areas per sample was calculated.

### Serum analysis

Blood samples were centrifuged to obtain serum. Serum concentrations of TRAcP 5b were measured using a commercially available assay (IDS), following the manufacturer’s protocol.

### RNA isolation and quantitative PCR analyses

Total RNA was extracted from tissues, cultured cells, or sorted cells using the RNeasy Plus Micro Kit (QIAGEN), TRIzol (MRC or Invitrogen) or GeneJET RNA purification kit (Thermo Fisher Scientific), following the manufacturers’ protocols. The RNA was quantified by NanoDrop spectrometry at 260 and 280 nm and complementary DNA was synthesized using either the iScript cDNA Synthesis Kit (BioRad) or High-Capacity RNA-to-cDNA Kit (Applied Biosystems). qPCR was performed by using the SsoFast EvaGreen Supermix (BioRad) or the TaqMan Fast Advanced Master Mix (Applied Biosystems) and gene-specific primers or TaqMan probes (see below) with the CFX384 Real time PCR detection system (BioRad), the ABI 7500 Fast system (Applied Biosystems) or the LightCycler 96 (Roche), following the manufacturers’ recommended procedures. For gene detection and quantification of murine genes, TaqMan probes and gene-specific primers were acquired from Thermo-Fisher Scientific, Invitrogen or TsingKe Biotech. The primers from Thermo-Fisher Scientific were the following: mouse *Nfatc1* (Mm01265944_m1); mouse *Ctsk* (Mm00484039_m1); mouse *Foxm1* (Mm00514924_m1); mouse *Oscar* (Mm01338227_g1); mouse *Mafb* (Mm00627481_s1); mouse *Nr4a1* (Mm01300401_m1); mouse *Irf8* (Mm00492567_m1); mouse *Il17a* (Mm00439618_m1); mouse *Il6* (Mm00446190_m1); mouse *Tnf* (Mm00443258_m1); mouse *Il1b* (Mm00434228_m1); mouse *Tnfsf11* (Mm00441906_m1) and mouse *Gapdh* (Mm99999915_g1). Custom primer sequences were: mouse *18s* (FW 5’-GTTCCGACCATAAACGATGCC-3’ and RV 5’-TGGTGGTGCCCTTCCGTCAAT -3’); mouse *Il17a* (FW 5’-AC CGCAATGAAGACCCTGAT-3’ and RV 5’-TCCCTCCGCATTGACACA-3’); mouse *Nfatc1* (FW 5’-GTTCCTTCAGCCAATCATCC-3’ and RV 5’-GGAGGTGATCTCGATTCTCG-3’); mouse *Il1b* (FW 5’-ATCCCAAGCAATACCCAAAG-3’ and RV 5’-GTGCTGATGTACC AGTTGGG-3’); mouse *Tnf* (FW 5’-AGCCCCCAGTCTGTATCCTTCT-3’ and RV 5’-AAGCCCATTTGAGTCCTTGATG-3’); mouse *Il6* (FW 5’-CCTTCCTACCCCAATTTCCAAT-3’ and RV 5’-AACGCACTAGGTTTGCCGAGTA-3’); mouse *Acp5* (FW 5’-CAGCCCTTACT ACCGTTTGC-3’ and RV 5’-GTAGTCCTCCTTGGCTGCTG-3’). Further primers used were mouse *Gapdh* (FW 5’-AGGTCGG TGTGAACGGATTTG-3’ and RV 5’-TGTAGACCATGTAGTTGAGGTCA-3’); mouse *Il6* (FW 5’-CTGCAAGAGACTTCCATCCAG-3’ and RV 5’-AGTGGTATAGACAGGTCTGTTGG-3’); mouse *Tnf* (FW 5’-CAGGCGGTGCCTATGTCTC-3’ and RV 5’-CGATCACCCC GAAGTTCAGTAG-3’); mouse *Nfatc1* (FW 5’-GGAGAGTCCGAGAATCGAGAT-3’ and RV 5’-TTGCAGCTAGGAAGTACGTCT-3’); mouse *Il1b* (FW 5’-ACGGACCCCAAAAGATGAAG -3’ and RV 5’-TTCTCCACAGCCACAATGAG-3’); mouse *Mitf* (FW 5’-CCAACA GCCCTATGGCTATGC-3’ and RV 5’-CTGGGCACTCACTCTCTGC-3’); mouse *Vav3* (FW 5’-ATGCAGACTCCAATTTCCATGAT-3’ and RV 5’-ATGGCCCTTGTTCAACGGAAT-3’); mouse *Rock2* (FW 5’-GGTTTACAGATGAAAGCGGAAGA-3’ and RV 5’-GTGATG CCTTATGACGAACCAA-3’); mouse *Myo5a* (FW 5’-GAAGTGTGGAAATCGGCAGAG-3’ and RV 5’-ATGTCAGGGTTCCGTAAGT GA-3’) and mouse *Hpgds* (FW 5’-AAGCTGACTGGCCTAAAATCAAG-3’ and RV 5’-CTCTGGTGGATTGTAAGTCCTTC-3’). Data were analyzed using the comparative (ΔΔCt) method and were normalized to *18s* or *Gapdh* mRNA. In LIP experiments gingival cyto-kine mRNA expression was assessed in ligated sites, the data are presented as fold change relative to the contralateral unligated control sites (baseline), set as 1.

### Single-cell RNA sequencing

CD45^+^CD11b^+^ synovial myeloid cells from hind paws of β-glucan or PBS pre-treated mice (7 days prior to K/BxN-STA induction) were sorted at day 17 after arthritis induction using a FACS Aria III sorter (BD, Germany). The cell concentration was adjusted to 1000–1600 cells per μl (1538 cells/μl in PBS sample and 1150 cells/μl in β-glucan sample). Approximately 10000 cells were loaded and then processed with Chromium Next GEM Single Cell 3’ v3.1 dual-index kit (10X Genomics) at the Dresden Concept Genome Center, Dresden, Germany. According to the manufacturer’s instructions, the cDNA was synthesized and subjected to library preparation and sequencing on the Novaseq 6000 platform (Illumina).

### Quantification and statistical analysis

#### Single-cell RNA sequencing

The raw sequencing data was processed with the ‘count’ command of the Cell Ranger software (v7.0.0) provided by 10X Genomics. To build the reference for Cell Ranger, mouse genome (GRCm39) as well as gene annotation (Ensembl 104) were downloaded from Ensembl. Genome and annotation were processed following the steps provided by 10x Genomics (https://support.10xgenomics.com/single-cell-gene-expression/software/release-notes/build#mm10_2020A) to build the appropriate Cell Ranger reference. All further data processing and analysis steps were performed in the R software environment using the package *Seurat* (v4.3). The data was filtered for low quality cells by excluding the libraries with less than 500 features and more than 10% mitochondrial reads. The data was normalized, and the most variable features selected using the function SCTransform. Appropriate functions for dimensionality reduction - principal component analysis (PCA) and Uniform Manifold Approximation and Projection (UMAP), with default *k nearest neighbor* parameters and resolution parameter set to 0.7 for the *FindClusters* function, were applied to the data and visualisations were produced. Cluster marker genes and differentially expressed genes were identified using appropriate functions from the *Seurat* package using the MAST and Wilcoxon rank-sum test, respectively. Multiple testing correction was performed using the Bonferroni correction. Differentially expressed genes were defined at FDR < 0.05 in the β-glucan group as compared to expression in the PBS control group. Log_2_ fold change ranked differentially expressed gene lists were used for GO enrichment analysis and significantly enriched ‘Molecular Functions’ and ‘Biological Process’ GO terms were defined by FDR < 0.05. All enrichment analyses were done using the *enrichR* R package (v3.2). Further visualisations were created using custom R functions based on the *ggplot2* package (v3.4.4).

#### Statistical analysis

The data are presented as mean±SEM. For the statistical comparisons of two groups, data were analyzed by a Student’s t-test or a Mann-Whitney U test, as appropriate. One-way ANOVA followed by Tukey’s multiple comparison test was used to compare more than two groups. For repeated-measurements, two-way ANOVA and Sidak’s multiple-comparisons test was used for data analysis. In Figure 4G, the Grubbs’ test was used to identify an outlier and resulted in the exclusion of one sample. All statistical analyses were conducted using GraphPad Prism software (GraphPad Inc., La Jolla, CA). P values < 0.05 were considered statistically significant.

## Supplementary Material

Supplementary file

## Highlights

- Trained immunity (TRIM) induced by β-glucan aggravates inflammatory bone loss
- β-glucan-induced TRIM promotes an osteoclastogenesis bias in the bone marrow
- TRIM promotes monocyte-mediated inflammatory osteoclastogenesis
- Trained monocyte transfer amplifies arthritis in recipients in an MITF-dependent way

## In brief

Haacke et al. show that induction of trained immunity by β-glucan is accompanied by adaptations in monocytes and bone marrow osteoclast precursors that drive an enhanced predisposition toward generation of osteoclasts, thereby leading to exacerbated inflammatory bone loss in experimental periodontitis and arthritis.
