# Supplementary material for: Innate immune training of osteoclastogenesis promotes inflammatory bone loss in mice
Source: Dev Cell. Author manuscript; Available in PMC 2025 Mar 28. (PMC7617534; doi:10.1016/j.devcel.2025.02.001)
Supplement: Supplementary file 1 [file EMS204031-supplement-Supplementary_file.pdf]

Developmental Cell, Volume *60*

Supplemental information

Innate immune training of osteoclastogenesis promotes inflammatory bone loss in mice

Nora Haacke, Hui Wang, Shu Yan, Marko Barovic, Xiaofei Li, Kosuke Nagai, Adelina Botezatu, Aikaterini Hatzioannou, Bettina Gercken, Giulia Trimaglio, Anisha U. Shah, Jun Wang, Ling Ye, Mangesh T. Jaykar, Martina Rauner, Ben Wielockx, Kyoung-Jin Chung, Mihai G. Netea, Lydia Kalafati, George Hajishengallis, and Triantafyllos Chavakis

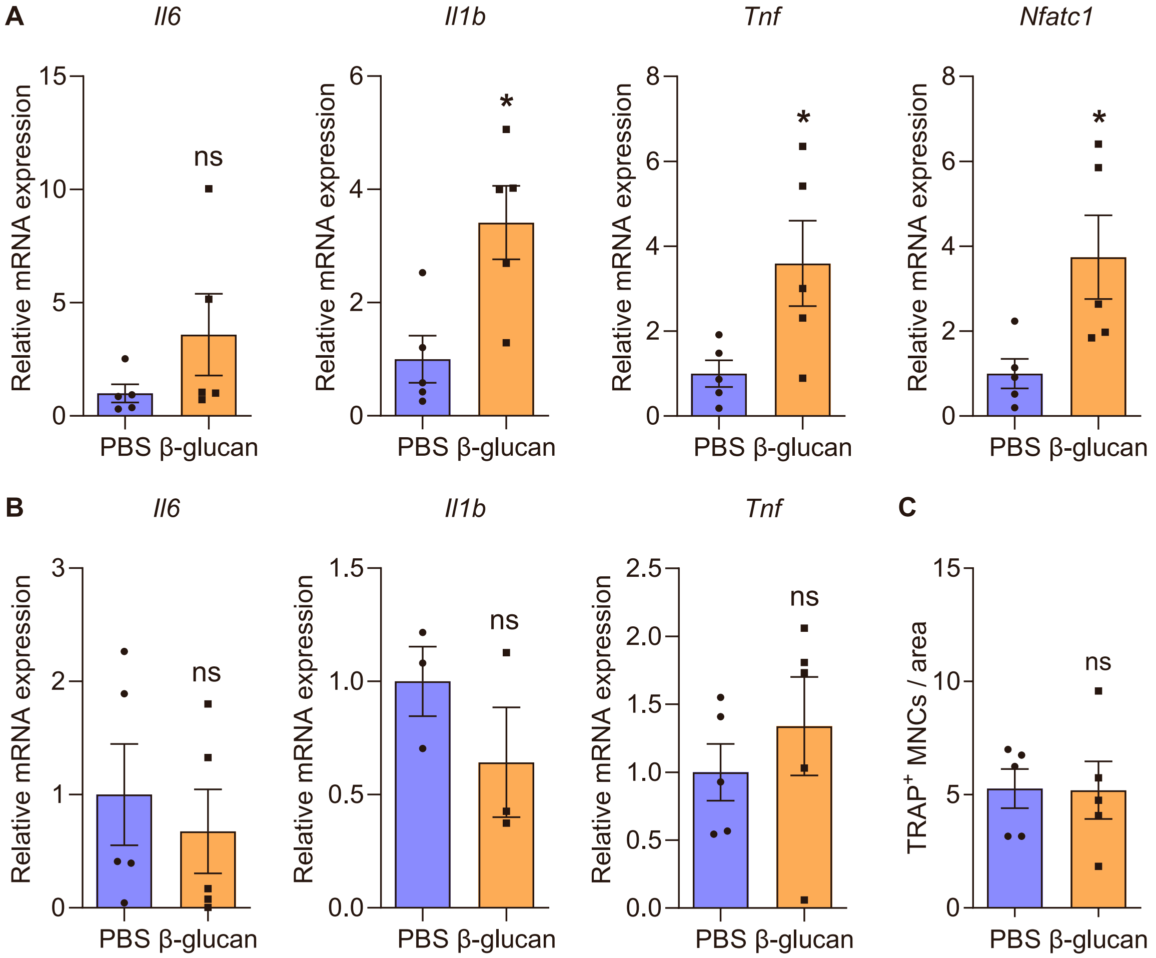

Figure S1. Gene expression of myeloid and stromal cells from the synovium of mice subjected to CAIA, related to Figure 2.

(A,B) Mice were pre-treated with β-glucan or PBS-control. After 7 days, both groups of mice were subjected to the CAIA model. On day 7 of the CAIA model, synovial $CD11b^+$ (A) and $CD45^-$ stromal cells (B) from the knee joints were isolated and processed for qPCR to measure relative mRNA expression of indicated molecules (n=3-5 mice per group). The results are presented relative to those of the PBS-control group, which was assigned an average value of 1.

(C) Mice were pre-treated with β-glucan or PBS-control and 7 days later, knee joints were collected and processed for TRAP staining; quantification of $TRAP^+$ MNCs per area (n= 5 mice per group) is shown.

Data are mean ± SEM; ns, non-significant; *$p<0.05$. Unpaired t-test (A-C). MNCs, multinucleated cells.

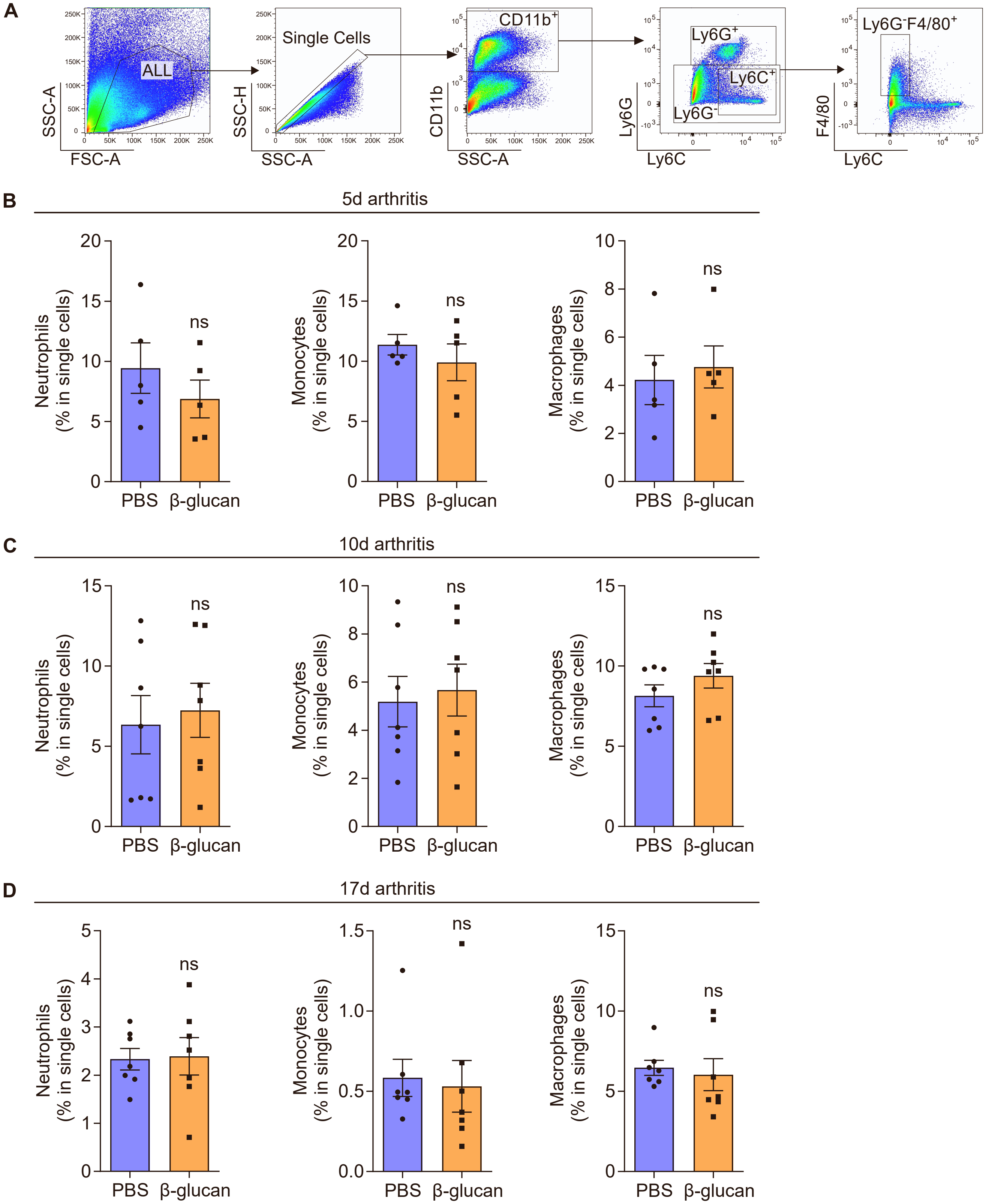

Figure S2. Effect of β-glucan-induced TRIM on innate immune cell populations in hind paws from mice subjected to K/BxN-STA, related to Figure 3.

Mice were pre-treated with β-glucan or PBS-control. After 7 days, both groups of mice were subjected to K/BxN-STA.

(A) Representative FACS plots from a PBS-control treated arthritic mouse (day 10) for the identification of neutrophils ($CD11b^{+}Ly6G^{+}$), monocytes ($CD11b^{+}Ly6G^{-}Ly6C^{+}$), and macrophages ($CD11b^{+}Ly6G^{-}F4/80^{+}$).

(B-D) Flow-cytometric analysis of neutrophils, monocytes and macrophages from hind paws at (B) day 5 (n= 5 mice per group), (C) day 10 (n= 7 mice per group) or (D) day 17 (n= 7 mice per group) after K/BxN-STA induction.

Data are mean ± SEM; ns, non-significant. Unpaired t-test (B-D), except for Monocytes in (D) (Mann-Whitney U-test).

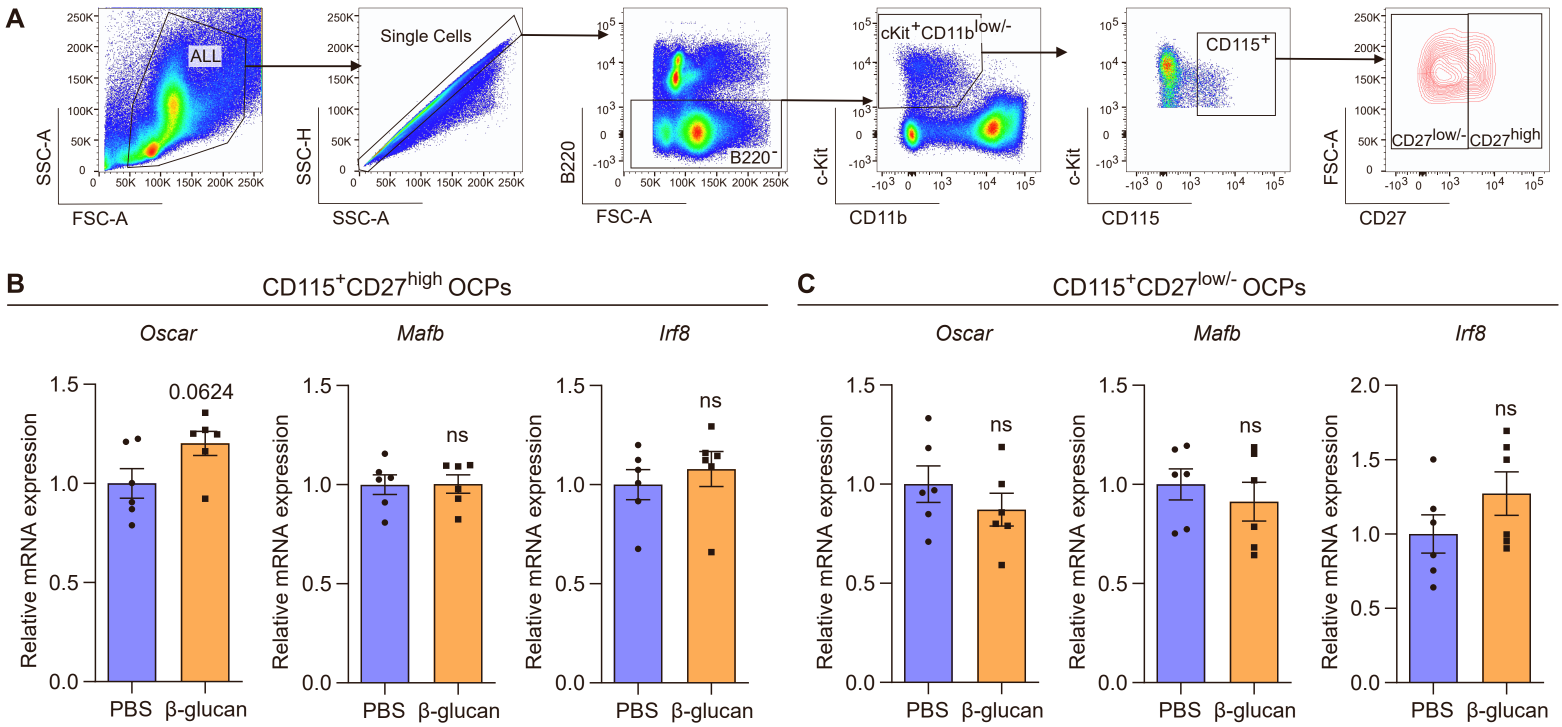

Figure S3. Effect of β-glucan-induced TRIM on OCPs in the bone marrow (BM), related to Figure 4.
(A) Representative FACS plots from a PBS-control treated mouse (0d - no arthritis) for the identification of $CD115^{+}CD27^{high}$ OCPs ($B220^{-}CD11b^{low/-}c\text{-}Kit^{+}CD115^{+}CD27^{high}$) and $CD115^{+}CD27^{low/-}$ OCPs ($B220^{-}CD11b^{low/-}c\text{-}Kit^{+}CD115^{+}CD27^{low/-}$) in the BM.
(B,C) Relative mRNA expression of the indicated molecules from sorted BM $CD115^{+}CD27^{high}$ OCPs
(B) and $CD115^{+}CD27^{low/-}$ OCPs (C) 7 days after β-glucan or PBS treatment (n= 6 mice per group). Results are presented relative to those of the PBS-control group, set as 1.
Data are mean ± SEM; ns, non-significant. Unpaired t-test (B,C), except for *Irf8* in (B) (Mann-Whitney U-test).

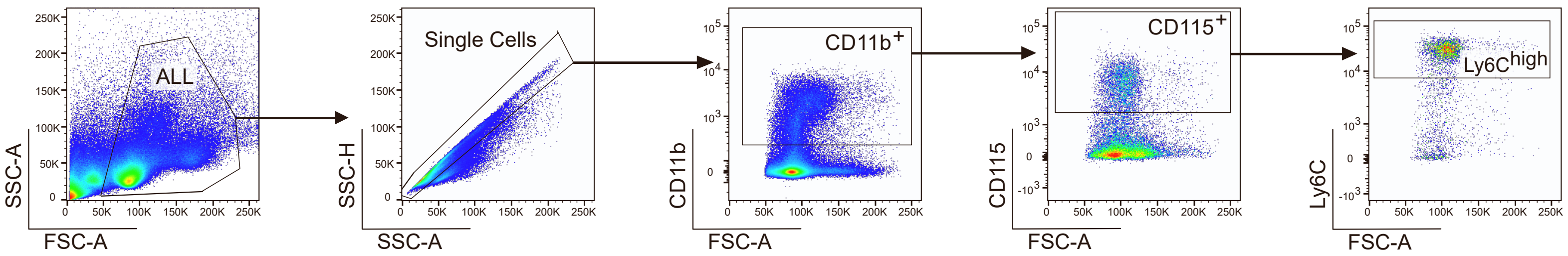

Figure S4. Flow cytometry analysis of classical monocytes, related to Figure 5.
Representative FACS plots from a PBS-control treated mouse (no arthritis) for the identification of classical monocytes ($CD11b^{+}CD115^{+}Ly6C^{high}$) from mouse spleen.

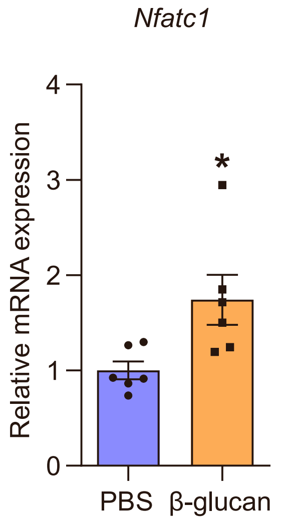

Figure S5. Trained Inflammatory osteoclastogenesis, related to Figure 5.
Mice were treated with β-glucan or PBS as control and after 7 days, splenic monocytes were isolated using the EasySep™ Mouse Monocyte Isolation Kit. Relative mRNA expression of *Nfatc1* was studied; results are presented as fold change relative to the PBS group, which was assigned an average value of 1. Data are mean ± SEM (n= 6 mice per group). *$p < 0.05$. Unpaired t-test.

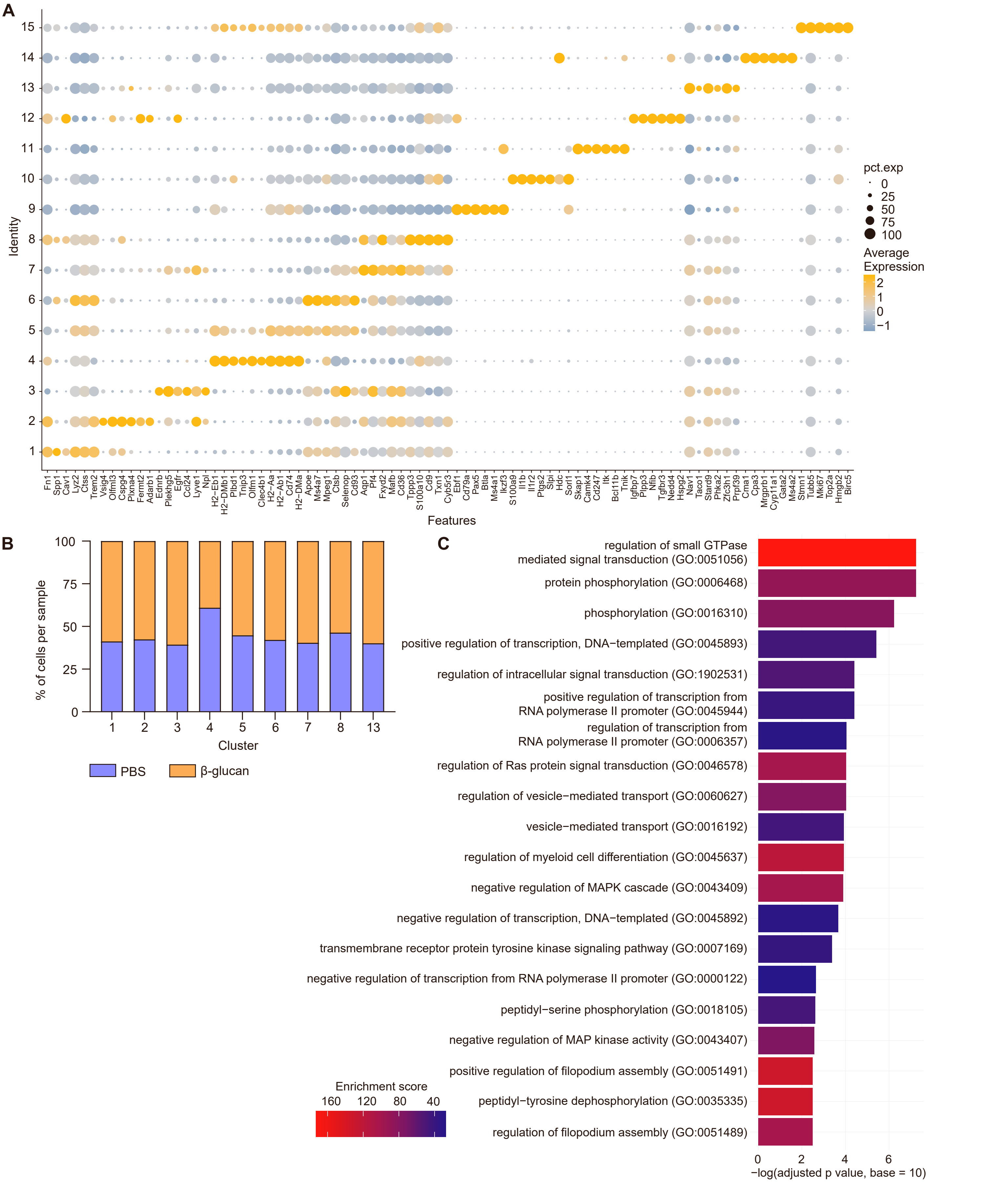

Figure S6. scRNA-seq from synovial myeloid cells, related to Figure 6.
(A-C) Mice were pre-treated with β-glucan or PBS and 7 days later subjected to K/BxN-STA for additional 17 days and myeloid cells ($CD45^{+}CD11b^{+}$) were sorted from the hind paws and scRNA-seq analysis was performed (n= 4 mice per group).
(A) Dot plot showing the top 6 marker genes for each cluster. 'pct.exp' displays the percentage of cells in the cluster expressing the gene. (B) Bar plot visualization of the distribution of cells within each of the clusters from the main myeloid cell compartment (clusters 1-8 and 13; see Figure 6A), normalized for the number of cells per sample in the dataset.
(C) Top 20 overrepresented GO terms of 'Biological Processes' from upregulated differentially expressed genes in the main myeloid cell compartment (clusters 1-8 and 13).

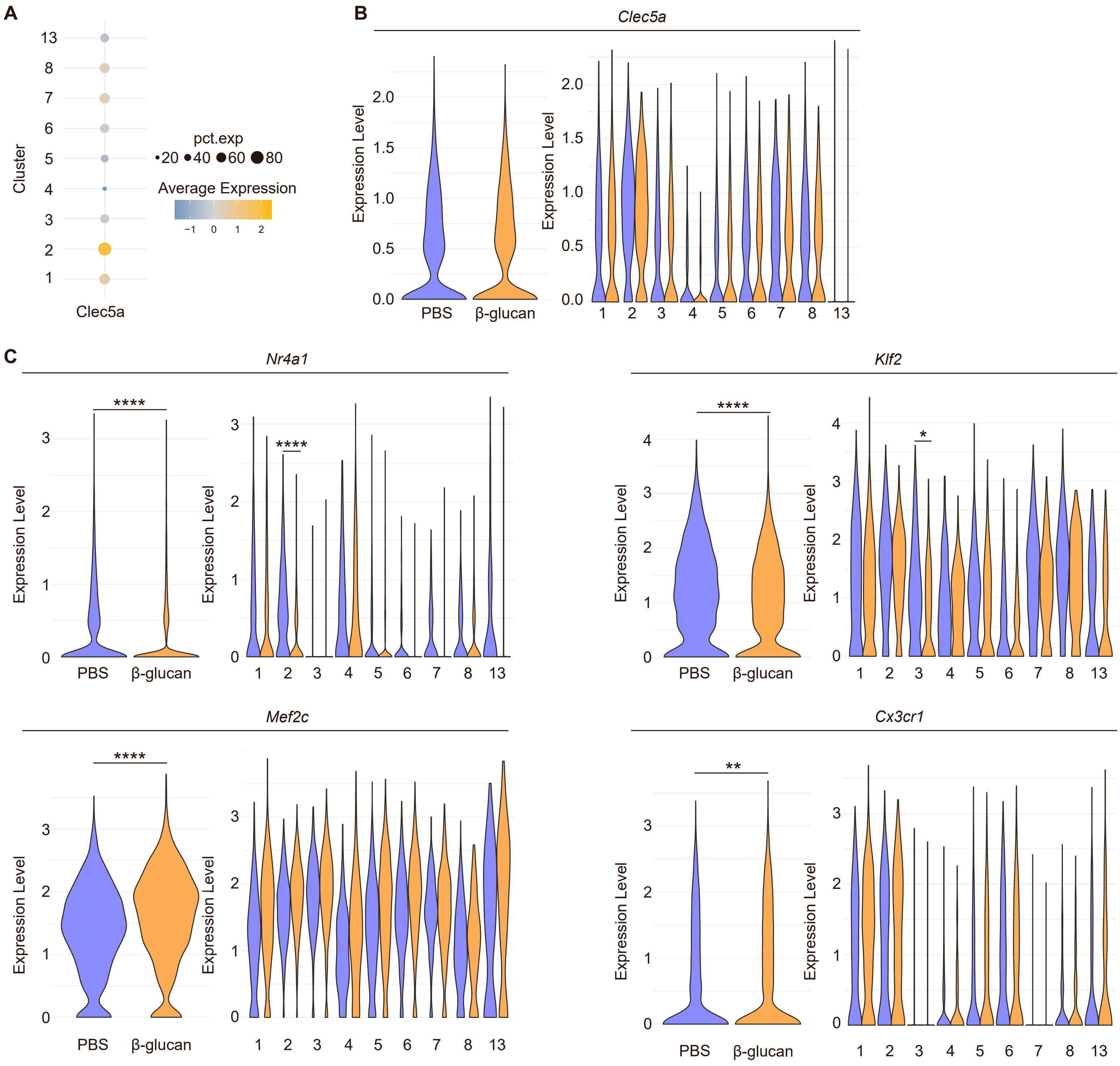

Figure S7. scRNA-seq from synovial myeloid cells and expression analysis of osteoclastogenesis-related genes, related to Figure 6.

Mice were pre-treated with β-glucan or PBS and 7 days later subjected to K/BxN-STA for additional 17 days and myeloid cells ($CD45^+CD11b^+$) were sorted from the hind paws and scRNA-seq analysis was performed (n= 4 mice per group).

(A) Dot plot showing gene expression of *Clec5a* for each cluster of the main myeloid cell compartment (comprising clusters 1-8 and 13). 'pct.exp' displays the percentage of cells in the cluster expressing the gene.

(B) Violin plots showing gene activity scores of *Clec5a* in the complete main myeloid cell compartment (clusters 1-8 and 13) (left panel) and in all separate clusters of the main myeloid cell compartment (right panel).

(C) Violin plots showing gene activity scores of *Nr4a1*, *Klf2*, *Mef2c* and *Cx3cr1* in the complete main myeloid cell compartment (clusters 1-8 and 13) (left panels) and in all separate clusters of the main myeloid cell compartment (right panels).

*FDR < 0.05; **FDR < 0.01; ****FDR < 0.0001. Wilcoxon rank-sum test with Bonferroni correction (B,C).

Supplemental tables

Table S1 (related to Figure 6): Enriched Gene Ontology (GO) 'Molecular Functions' terms for upregulated differentially expressed genes.

Term	In.List	In.Annotation	p_val	p_val_adj	Combined.Score	Genes
GTPase activator activity (GO:0005096)	33	249	3,85394E-15	1,8576E-12	211,2367524	NRP1;DOCK4;RABGAP1;RAP1GDS1;AGAP1;ARHGAP18;ARHGAP17;ASAP1;RASAL2;ARHGAP15;ARHGAP12;RASGRP3;ARHGAP22;RGS2;ADAP2;OPHN1;STXBP5;SRGAP2;TBC1D22A;EVI5;VAV3;RABGAP1L;STARD8;MYO9B;MYO9A;ARHGAP24;ARHGAP45;ACAP2;RASA3;TBC1D5;GNAQ;RASA1;CDC42EP3
GTPase regulator activity (GO:0030695)	34	275	1,19118E-14	2,87074E-12	188,5924239	NRP1;DOCK4;RABGAP1;RAP1GDS1;AGAP1;ARHGAP18;ARHGAP17;ASAP1;RASAL2;ARHGAP15;ARHGAP12;RASGRP3;ARHGAP22;RGS2;ADAP2;OPHN1;STXBP5;SRGAP2;TBC1D22A;EVI5;VAV3;RABGAP1L;STARD8;MYO9B;MYO9A;ARHGAP24;ARHGAP45;ACAP2;RASA3;TBC1D5;GNAQ;RASA1;CDC42EP3;RIN2
protein serine/threonine kinase activity (GO:0004674)	28	368	1,69736E-07	2,72709E-05	52,57003798	SMG1;CAMK2D;ROCK2;STK3;RPS6KA3;NUAK1;AKT3;STK38;MAP3K20;MAP4K3;MAP2K5;MAP4K4;MAP3K5;MAP3K3;CDK19;PRKCH;PRKCB;NEK6;NEK7;VRK2;MAPK14;ERN1;CDK6;TAOK1;BMP2K;ULK2;SGK3;CSNK1G1
Rho guanyl-nucleotide exchange factor activity (GO:0005089)	10	59	1,69866E-06	0,000204688	108,538148	EPS8;VAV3;FGD3;AKAP13;FGD4;ARHGEF12;PLEKHG5;ITSN1;ARHGEF3;ARHGEF10L
protein kinase activity (GO:0004672)	31	513	5,25736E-06	0,00050681	31,98190357	CSF1R;SMG1;CAMK2D;ROCK2;STK3;RPS6KA3;NUAK1;CUX1;AKT3;STK38;MAP3K20;MAP2K5;MAP4K3;MAP3K5;MAP4K4;MAP3K3;PRKCH;PRKCB;NEK6;NEK7;VRK2;MERTK;MAPK14;ERN1;FER;PEAK1;TAOK1;BMP2K;ULK2;SGK3;CSNK1G1
phosphatidylinositol binding (GO:0035091)	12	100	6,88503E-06	0,000553097	65,02114422	DENND1B;MYO1E;HIP1;PLEKHA2;DENND1A;FCHO2;TULP4;SNX13;WDFY3;SNX10;SNX30;MTM1
Rab guanyl-nucleotide exchange factor activity (GO:0017112)	8	51	3,3731E-05	0,002322617	76,39792551	DENND1B;DENND1A;DENND4C;DENND5A;DENND6A;GAPVD1;SBF2;RIN2
protein kinase binding (GO:0019901)	28	495	4,87793E-05	0,00293895	24,21388962	PRKN;CSF1R;CLTC;PLEK;PRKAG2;PTPRJ;NR3C1;HDAC9;SPRED1;RB1CC1;STK38;KIF13B;SASH1;NSF;PTPN1;DUSP3;PPP1R12A;PRKCB;NEK6;AP1B1;VRK2;MERTK;KAT2B;GPRC5B;NBR1;ATF7;RHOQ;LIMS1
Ras GTPase binding (GO:0017016)	15	184	5,76296E-05	0,003086386	34,82723981	NSF;DENND1B;RABGAP1L;RABGAP1;DENND1A;MYO5A;RANBP9;MYO9B;AKAP13;TBC1D5;EXOC4;STXBP5;TBC1D22A;EVI5;RAPGEF6
transcription regulatory region DNA binding (GO:0044212)	23	374	6,60933E-05	0,003185696	25,53712587	RB1;MEF2A;PRKN;HDAC4;MEF2C;TCF12;JMJD1C;ARID5B;MITF;IKZF1;NR3C1;TRERF1;ST18;ELK3;RUNX1;KANSL1;KLHL6;TBL1XR1;EP300;TCF4;TBL1X;RREB1;NFE2L2
GTPase binding (GO:0051020)	9	76	0,000107665	0,004717669	48,98321968	FGD3;FGD4;ACAP2;ADAP2;RASA1;AGAP1;ASAP1;AMBRA1;FNBP1L
protein tyrosine phosphatase activity (GO:0004725)	8	62	0,000141135	0,005517152	52,34965253	PTPN1;DUSP3;UBASH3B;PTPRA;EYA4;PTPRJ;DUSP6;DUSP7
phosphoprotein phosphatase activity (GO:0004721)	12	136	0,000148803	0,005517152	34,14964823	PPP3CA;PTPN1;DUSP3;UBASH3B;PTPRA;EYA4;PPM1H;PTPRJ;SSH2;DUSP6;MTM1;DUSP7
activating transcription factor binding (GO:0033613)	8	68	0,000271044	0,009331645	43,63334948	RB1;MEF2A;HDAC4;MEF2C;CREBBP;NEK6;EP300;NFE2L2
Rho GTPase binding (GO:0017048)	8	72	0,000402588	0,012936479	38,92779335	EPS8;CYFIP1;AKAP13;DOCK4;CDC42EP3;ARHGAP17;MYO9B;SRGAP2
AP-2 adaptor complex binding (GO:0035612)	3	7	0,000492587	0,014839181	225,926277	HIP1;TBC1D5;BMP2K
low-density lipoprotein particle binding (GO:0030169)	4	17	0,000687933	0,018421308	88,76169225	COLEC12;MSR1;STAB1;CD36
ATP binding (GO:0005524)	16	255	0,0006678	0,018421308	19,6452549	NEK6;PRKAG2;MYO9B;IDE;STK3;ERN1;P2RX7;CDK6;TAOK1;AKT3;STK38;MAP3K20;MAP4K3;MYO1F;MAP4K4;MAP3K5
microfilament motor activity (GO:0000146)	4	18	0,000867261	0,021162901	79,79548291	MYO1E;MYO10;MYO5A;MYO9B
small GTPase binding (GO:0031267)	7	63	0,000922035	0,021162901	34,74454422	FGD3;FGD4;ACAP2;ADAP2;AGAP1;ASAP1;RAPGEF6
phosphatidylinositol phosphate binding (GO:1901981)	8	81	0,000891314	0,021162901	30,64457684	DENND1B;OSBPL8;HIP1;ADAP2;MYO10;DENND1A;SNX13;OGT
RNA polymerase II activating transcription factor binding (GO:0001102)	6	47	0,00102329	0,022419353	40,0191776	RB1;MEF2A;CREBBP;MEF2C;EP300;NFE2L2
MAP kinase phosphatase activity (GO:0033549)	3	9	0,001139137	0,023872351	134,0237202	DUSP3;DUSP6;DUSP7
histone deacetylase binding (GO:0042826)	8	85	0,001223784	0,024577668	27,73551609	MEF2A;PRKN;HDAC4;KAT2B;NIPBL;MEF2C;MIER1;HDAC9
histone acetyltransferase activity (H4-K8 specific) (GO:0043996)	3	10	0,001597474	0,024865684	109,1402338	KANSL1;PHF20;OGT
histone acetyltransferase activity (H4-K5 specific) (GO:0043995)	3	10	0,001597474	0,024865684	109,1402338	KANSL1;PHF20;OGT
histone acetyltransferase activity (H4-K16 specific) (GO:0046972)	3	10	0,001597474	0,024865684	109,1402338	KANSL1;PHF20;OGT
mitogen-activated protein kinase binding (GO:0051019)	4	20	0,001320271	0,024865684	65,65235926	NBR1;PTPRJ;ATF7;MAPK14
scavenger receptor activity (GO:0005044)	4	21	0,001599245	0,024865684	60,00079433	COLEC12;MSR1;STAB1;CD36
SH3/SH2 adaptor activity (GO:0005070)	6	51	0,00157913	0,024865684	34,15719684	BCAR3;EPS8;VAV3;SH3BGRL;BLNK;SKAP2
Rab GTPase binding (GO:0017137)	10	130	0,001508502	0,024865684	21,59307876	NSF;DENND1B;RABGAP1L;RABGAP1;TBC1D5;DENND1A;MYO5A;STXBP5;TBC1D22A;EVI5
adenyl ribonucleotide binding (GO:0032559)	16	279	0,001712617	0,025796291	15,53359043	NEK6;PRKAG2;MYO9B;IDE;STK3;ERN1;P2RX7;CDK6;TAOK1;AKT3;STK38;MAP3K20;MAP4K3;MYO1F;MAP4K4;MAP3K5
kinase binding (GO:0019900)	21	418	0,00187165	0,027337434	13,36447712	RB1;PRKN;NSF;PTPN1;DUSP3;PPP1R12A;NEK6;CLTC;AP1B1;PRKAG2;PTPRJ;VRK2;NR3C1;KAT2B;GPRC5B;SPRED1;RB1CC1;KIF13B;RHOQ;SASH1;LIMS1
lipoprotein particle binding (GO:0071813)	4	23	0,002275228	0,032254696	50,74000063	COLEC12;MSR1;STAB1;CD36
phosphatidylinositol-3,4-bisphosphate binding (GO:0043325)	4	24	0,00267738	0,032591039	46,91142579	HIP1;ADAP2;PLEKHA2;PLEK
acetyltransferase activity (GO:0016407)	4	24	0,00267738	0,032591039	46,91142579	KAT2B;CREBBP;KAT6A;EP300
peptide-lysine-N-acetyltransferase activity (GO:0061733)	5	39	0,002639926	0,032591039	34,62126393	KAT2B;CREBBP;KAT6A;EP300;EPC1
transferase activity, transferring acyl groups other than amino-acyl groups (GO:0016747)	6	56	0,002566035	0,032591039	28,42059584	KAT2B;CREBBP;CERS6;ZDHHC20;KAT6A;EP300
transcription coactivator activity (GO:0003713)	16	291	0,002614397	0,032591039	13,86054737	RB1;MEF2A;NCOA2;WWOX;CREBBP;PRKCB;ARID5B;ARID1A;TRERF1;ARID1B;PIAS1;KAT2B;KAT6A;EP300;NCOA7;LPIN1
transcription regulatory region sequence-specific DNA binding (GO:0000976)	16	292	0,002704651	0,032591039	13,73079503	MEF2A;PRKN;MEF2C;CREBBP;ZFHX3;ZMYND8;JMJD1C;ARID5B;PHF21A;RUNX1;CUX1;KLHL6;IRF2;CREBRF;RREB1;NFE2L2
core promoter binding (GO:0001047)	8	97	0,002841936	0,033410081	20,96801988	RB1;HDAC4;ZFHX3;KANSL1;EP300;NR3C1;ST18;RUNX1
RNA polymerase II transcription factor binding (GO:0001085)	9	121	0,003211657	0,03685759	18,36975104	RB1;MEF2A;RERE;HDAC4;MEF2C;CREBBP;EP300;RBPJ;NFE2L2
histone acetyltransferase activity (GO:0004402)	5	41	0,003299759	0,036987993	31,46592004	KAT2B;CREBBP;KAT6A;EP300;EPC1
platelet-derived growth factor receptor binding (GO:0005161)	3	13	0,003602027	0,039317118	66,74135052	ERN1;PDGFC;PTPRJ
androgen receptor binding (GO:0050681)	5	42	0,003670685	0,039317118	30,0431569	RB1;PRKCB;EP300;PIAS1;FOXP1
Rac GTPase binding (GO:0048365)	5	43	0,004070571	0,042652504	28,71163017	EPS8;CYFIP1;DOCK4;ARHGAP17;SRGAP2

kinase activity (GO:0016301)	15	280	0,004458888	0,045727316	12,25488592	MAP3K3;SMG1;DGKD;DGKZ;STK3;RPS6KA3;PIK3CA;PEAK1;TAOK1;AKT3;SGK3;MAP4K3;MAP2K5;MAP4K4;MAP3K5
phosphotransferase activity, alcohol group as acceptor (GO:0016773)	14	254	0,004611047	0,046302599	12,54128432	MAP3K3;SMG1;DGKD;DGKZ;STK3;RPS6KA3;PIK3CA;PEAK1;TAOK1;AKT3;SGK3;MAP4K3;MAP2K5;MAP3K5
purine ribonucleoside triphosphate binding (GO:0035639)	19	396	0,004947101	0,04866332	10,72876305	NEK6;PRKAG2;MYO9B;IDE;STK3;P2RX7;ERN1;CDK6;RAP2B;RAB14;TAOK1;AKT3;STK38;MAP3K20;MYO1F;RHOQ;MAP4K3;MAP3K5 ;MAP4K4

Table S1 (related to Figure 6): Enriched Gene Ontology (GO) 'Molecular Functions' terms for upregulated differentially expressed genes.

Results from Gene Ontology (GO) enrichment analysis of 'Molecular Functions' based on scRNA-seq data from synovial myeloid cells ($CD45^{+}CD11b^{+}$) from mice pre-treated with β-glucan or PBS and 7 days later subjected to K/BxN-STA for 17 days. Enrichment analysis was performed with upregulated differentially expressed genes (ranked according to Log_2 fold change) in the main myeloid cell compartment comprising the clusters 1-8 and 13 (see Figure 6A). Significantly enriched GO terms were defined by FDR < 0.05. 'Term' is the functional annotation in the database; 'In.List' is the number of genes in the list of interest with this functional annotation; 'In.Annotation' is the total number of genes with this functional annotation; 'p_val' is the uncorrected p-value; 'p_val_adj' is the p-value adjusted for multiple testing; 'Combined.Score' is the enrichment score as reported by enrichr; 'Genes' are the genes in the differentially expressed gene list that are contained in the functional annotation.

Table S2 (related to Figure SF6): Enriched Gene Ontology (GO) 'Biological Process' terms for upregulated differentially expressed genes.

Term	In.List	In.Annotation	p_val	p_val_adj	Combined.Score	Genes
regulation of small GTPase mediated signal transduction (GO:0051056)	21	140	3,88998E-11	5,9473E-08	172,6287522	VAV3;ARHGEF12;STARD8;PLEKHG5;ITSN1;ARHGAP18;ARHGAP17;MYO9B;ARHGAP15;MYO9A;ARHGAP24;ARHGAP45;ARHGAP12;ARHGAP22;FGD3;AKAP13;FGD4;OPHN1;ARHGEF3;SRGAP2;RHOQ
protein phosphorylation (GO:0006468)	39	470	4,74645E-11	5,9473E-08	89,77146594	CSF1R;SMG1;RNASEL;CAMK2D;ROCK2;STK3;RPS6KA3;NUAK1;AKT3;STK38;MAP3K20;FNIP2;MAP4K3;MAP3K5;MAP4K4;MAP3K3;CDK19;PRKCH;PRKCB;NEK6;NEK7;VRK2;MERTK;MAPK14;PHKA2;ERN1;FER;CDK6;TEC;PIK3CA;PEAK1;TAOK1;ABI1;BMP2K;ULK2;BIRC6;SGK3;TLR4;CSNK1G1
phosphorylation (GO:0016310)	33	386	7,20235E-10	6,01636E-07	81,40134398	RNASEL;CAMK2D;DGKD;ROCK2;STK3;RPS6KA3;NUAK1;AKT3;STK38;MAP3K20;FNIP2;MAP4K3;MAP3K5;MAP4K4;CDK19;PRKCH;PRKCB;NEK6;NEK7;VRK2;MERTK;PHKA2;DGKZ;ERN1;FER;CDK6;TEC;PIK3CA;PEAK1;TAOK1;BMP2K;BIRC6;SGK3
positive regulation of transcription, DNA-templated (GO:0045893)	61	1120	6,26158E-09	3,92288E-06	46,1347756	RB1;PID1;DDX3X;IKZF1;RBPJ;NR3C1;ELK3;RPS6KA3;PPP3CA;ZMIZ1;EPC1;EP300;MAP3K5;MEF2A;BCAS3;NCOA2;MEF2C;PPP1R12A;TCF12;MITF;ETV1;ARID1A;ARID1B;PIAS1;RUNX1;KAT2B;ZEB2;MDFIC;TBL1XR1;KAT6A;TET3;IRF2;IRF8;NCOA7;TLR4;RHOQ;HDAC4;PRKN;RNASEL;TNNI2;TBL1X;RREB1;MAP2K5;BPTF;ATF7IP;WWOX;CREBBP;ZFHX3;TRERF1;FLI1;ST18;MLLT10;DAB2;KLHL6;ASXL2;CREBRF;TCF4;LPIN1;OGT;PF4;NFE2L2
regulation of intracellular signal transduction (GO:1902531)	31	422	8,14833E-08	3,94016E-05	53,1933464	PHF20;ITSN1;EHMT1;PRKAG2;ARHGAP18;ARHGAP17;ARHGAP15;ARHGAP12;FGD3;ARHGAP22;FGD4;AKAP13;NUAK1;SPRED1;OPHN1;PIP4K2A;EP300;SRGAP2;VAV3;ARHGEF12;STARD8;PLEKHG5;MYO9B;VRK2;MAPK14;MYO9A;ARHGAP24;ARHGAP45;KAT6A;ARHGEF3;RHOQ
positive regulation of transcription from RNA polymerase II promoter (GO:0045944)	48	848	9,43374E-08	3,94016E-05	40,52185199	HDAC4;PRKN;RNASEL;PID1;DDX3X;ARHGEF10L;IKZF1;RBPJ;NR3C1;ELK3;RPS6KA3;PPP3CA;NIPBL;ZMIZ1;EPC1;EP300;TBL1X;RREB1;MAP2K5;MEF2A;WWOX;BCAS3;NCOA2;MEF2C;CREBBP;PPP1R12A;TCF12;MITF;ETV1;ST18;RUNX1;MLLT10;KAT2B;ZEB2;TBL1XR1;TET3;ASXL2;IRF2;CREBRF;IRF8;NCOA7;TCF4;LPIN1;OGT;TLR4;RHOQ;PF4;NFE2L2
regulation of transcription from RNA polymerase II promoter (GO:0006357)	69	1478	2,57766E-07	9,22802E-05	31,48394497	RB1;PID1;DDX3X;IKZF1;RBPJ;NR3C1;BACH1;ELK3;RPS6KA3;PPP3CA;NIPBL;ZMIZ1;NFKBIZ;EPC1;EP300;MEF2A;BCAS3;NCOA2;MEF2C;PPP1R12A;PRKCB;TCF12;ARID5B;MITF;ETV1;ARID1A;FOXP1;RUNX1;KAT2B;ZEB2;TBL1XR1;TET3;IRF2;IRF8;NCOA7;TLR4;RHOQ;HDAC4;PRKN;RNASEL;CAMK2D;NEDD4L;HDAC9;PHF21A;CUX1;APOE;TBL1X;FNIP1;RREB1;FNIP2;MAP2K5;BPTF;WWOX;CREBBP;ZFHX3;MAPK14;TRERF1;FLI1;ST18;MLLT10;BCL6;KLHL6;ASXL2;CREBRF;TCF4;LPIN1;OGT;PF4;NFE2L2
regulation of Ras protein signal transduction (GO:0046578)	13	90	3,30959E-07	9,30554E-05	101,3251033	DENND1A;AGAP1;ASAP1;ARHGAP17;MYO9B;RASAL2;EPS8;ACAP2;ADAP2;OPHN1;RASA3;RASA1;OGT
regulation of vesicle-mediated transport (GO:0060627)	16	138	3,34197E-07	9,30554E-05	78,96594172	NSF;PRKN;NRP1;PTPN1;RABGAP1L;HIP1;RABGAP1;DNAJC13;OPHN1;RAB40C;RAB14;TBC1D5;BMP2K;STXBP5;TBC1D22A;EVI5
vesicle-mediated transport (GO:0016192)	29	410	4,7207E-07	0,000118301	45,40007754	DENND1B;DENND1A;CLTC;AGAP1;SNX10;ASAP1;FNBP1L;SNX30;MTM1;ADAP2;CUX1;VTI1A;VPS54;DENND5A;EVI5;EPS15;NSF;BCAS3;MYO5A;VRK2;MYO1E;ACAP2;EHD4;RABEP1;RAB14;TBC1D5;SPIRE1;RHOQ;CSNK1G1
regulation of myeloid cell differentiation (GO:0045637)	11	65	5,22046E-07	0,000118932	118,170899	KAT2B;MEF2C;CREBBP;CDK6;AGO3;PRKCB;KMT2C;EP300;RUNX1;PF4;TNRC6B
negative regulation of MAPK cascade (GO:0043409)	12	80	6,18247E-07	0,000129111	101,3117655	P2RX7;PTPN1;SPRED1;RGS2;DUSP3;STK38;RANBP9;PTPRJ;APOE;TLR4;DUSP6;DUSP7
negative regulation of transcription, DNA-templated (GO:0045892)	44	813	1,11963E-06	0,000215829	32,49689859	RB1;HDAC4;PRKN;EHMT1;NEDD4L;IKZF1;RBPJ;NR3C1;BACH1;HDAC9;PHF21A;ELK3;NIPBL;BTAF1;ATXN1;CUX1;EPC1;EP300;TBL1X;FNIP1;RREB1;FNIP2;MAP2K5;BPTF;MEF2A;ATF7IP;WWOX;TSC22D4;MEF2C;CREBBP;ZFHX3;WWP1;ARID5B;MITF;FOXP1;ZEB2;DAB2;BCL6;MDFIC;TBL1XR1;KAT6A;IRF2;IRF8;LIMS1
transmembrane receptor protein tyrosine kinase signaling pathway (GO:0007169)	27	396	2,36444E-06	0,000423235	38,67491685	ATP6V1A;CYFIP1;CSF1R;NRP1;ROCK2;ITSN1;NEDD9;PTPRJ;IDE;SPRED1;UBASH3B;PDGFC;BLNK;ATP6V0A1;VAV3;CYBB;MAPK14;MTSS1;FER;TEC;PIK3CA;RAB14;RASA1;ABI1;ELMO1;LCP2;RHOQ
negative regulation of transcription from RNA polymerase II promoter (GO:0000122)	32	565	1,38615E-05	0,002315791	27,45903332	RB1;HDAC4;PRKN;ZMYND8;NEDD4L;RBPJ;NR3C1;BACH1;HDAC9;PHF21A;NIPBL;CUX1;EPC1;EP300;TBL1X;FNIP1;RREB1;FNIP2;MAP2K5;BPTF;MEF2A;WWOX;MEF2C;CREBBP;ZFHX3;ARID5B;MITF;ZEB2;BCL6;TBL1XR1;IRF2;IRF8
peptidyl-serine phosphorylation (GO:0018105)	14	145	1,55546E-05	0,002436243	47,55535442	SMG1;CAMK2D;PRKCH;PRKCB;ROCK2;NEK6;VRK2;MAPK14;ERN1;RPS6KA3;AKT3;STK38;SGK3;CSNK1G1
negative regulation of MAP kinase activity (GO:0043407)	9	61	1,82006E-05	0,002682975	75,4502617	PTPN1;SPRED1;RGS2;DUSP3;STK38;PTPRJ;APOE;DUSP6;DUSP7
positive regulation of filopodium assembly (GO:0051491)	6	25	2,67624E-05	0,003193649	132,2114191	NRP1;RIPOR2;DOCK11;BCAS3;FNBP1L;RHOQ
peptidyl-tyrosine dephosphorylation (GO:0035335)	6	25	2,67624E-05	0,003193649	132,2114191	PTPN1;DUSP3;UBASH3B;PTPRJ;DUSP6;DUSP7
regulation of filopodium assembly (GO:0051489)	7	36	2,47424E-05	0,003193649	101,9672861	NRP1;RIPOR2;DOCK11;BCAS3;MYO10;FNBP1L;RHOQ
regulation of megakaryocyte differentiation (GO:0045652)	8	49	2,49491E-05	0,003193649	82,4797912	KAT2B;MEF2C;AGO3;KMT2C;EP300;RUNX1;PF4;TNRC6B
regulation of lipid metabolic process (GO:0019216)	11	100	3,78789E-05	0,004314747	50,37253092	RB1;NCOA2;CPT1A;CREBBP;TBL1XR1;CHD9;PRKAG2;PLIN2;CD36;TBL1X;RGL1
platelet-derived growth factor receptor signaling pathway (GO:0048008)	5	17	4,44433E-05	0,004632807	165,762986	PTPN1;NRP1;FER;PDGFC;PTPRJ
regulation of endocytosis (GO:0030100)	9	69	4,99145E-05	0,004632807	59,32135979	PTPN1;DAB2;MCTP1;HIP1;OPHN1;RAB14;BMP2K;NEDD4L;APOE
positive regulation of plasma membrane bounded cell projection assembly (GO:0120034)	9	69	4,99145E-05	0,004632807	59,32135979	EPS8;P2RX7;RIPOR2;NRP1;DOCK11;BCAS3;CDC42EP3;FNBP1L;RHOQ
positive regulation of gene expression (GO:0010628)	38	771	4,88466E-05	0,004632807	21,09171758	RB1;HDAC4;PRKN;PID1;DDX3X;ROCK2;TNNI2;EPC1;EP300;CD36;TBL1X;RREB1;MAP3K5;BPTF;ATF7IP;MEF2C;CREBBP;ZFHX3;MITF;ARID1A;MAPK14;TRERF1;FLI1;PIAS1;RUNX1;P2RX7;ERN1;KAT2B;DAB2;MDFIC;KLHL6;TBL1XR1;KAT6A;TCF4;TLR4;LIMS1;PF4;NFE2L2
cellular protein modification process (GO:0006464)	46	1001	4,41166E-05	0,004632807	19,91159049	RNASEL;CAMK2D;ROCK2;TULP4;F13A1;MTM1;STK3;FBXL20;RPS6KA3;PPP3CA;NUAK1;AKT3;STK38;MAP3K20;ST8SIA4;APOE;FNIP2;MAP4K3;MAP3K5;MAP4K4;PTPN1;CDK19;PRKCH;PPP1R12A;PRKCB;FBXW7;NEK6;NEK7;VRK2;MERTK;SSH2;PHKA2;DUSP6;DUSP7;KLHL9;ERN1;FER;CDK6;TEC;PIK3CA;PEAK1;TAOK1;BMP2K;BIRC6;SGK3;FBXL5
vascular endothelial growth factor receptor signaling pathway (GO:0048010)	9	70	5,60388E-05	0,005015469	57,6641685	VAV3;CYFIP1;NRP1;PIK3CA;ROCK2;ABI1;ELMO1;CYBB;MAPK14

negative regulation of protein localization to plasma membrane (GO:1903077)	5	19	8,01556E-05	0,006086968	133,7072515	DAB2;PID1;CLTC;NUMB;RHOQ
negative regulation of protein localization to cell periphery (GO:1904376)	5	19	8,01556E-05	0,006086968	133,7072515	DAB2;PID1;CLTC;NUMB;RHOQ
regulation of Rho protein signal transduction (GO:0035023)	8	57	7,68576E-05	0,006086968	61,66210857	EPS8;RIPOR2;NRP1;AKAP13;OPHN1;LPAR6;MYO9B;APOE
regulation of GTPase activity (GO:0043087)	15	188	7,36427E-05	0,006086968	33,16056196	VAV3;BCAS3;RABGAP1;DOCK8;RAP1GDS1;SNX13;DOCK10;FGD3;DOCK11;FGD4;LRCH1;SRGAP2;EVI5;RAPGE F6;LIMS1
activation of protein kinase activity (GO:0032147)	17	233	7,64687E-05	0,006086968	30,03947867	MAP3K3;OSBPL8;KIDINS220;MAPK14;DUSP6;DUSP7;STK3;ADCY9;TAOK1;MAP3K20;S1PR2;TLR4;PIBF1;MAF 4K3;MAP2K5;MAP4K4;MAP3K5
post-Golgi vesicle-mediated transport (GO:0006892)	8	58	8,7245E-05	0,006430469	59,61720039	RAB14;VPS13C;EXOC4;VTI1A;EXOC6B;VPS54;MYO5A;EPS15
regulation of focal adhesion assembly (GO:0051893)	7	44	9,57095E-05	0,006852798	69,69875975	NRP1;MACF1;BCAS3;ROCK2;PTPRJ;CLASP2;LIMS1
phosphatidylinositol metabolic process (GO:0046488)	11	112	0,00010754	0,007485967	39,81373694	INPP4A;CSF1R;SMG1;PIK3CA;RAB14;PLEKHA2;PLEK;PIP4K2A;PLA2G4A;FAM126A;MTM1
positive regulation of cell-matrix adhesion (GO:0001954)	6	33	0,00014146	0,009328936	78,29216362	NRP1;CDK6;PLEKHA2;PTPRJ;CD36;LIMS1
positive regulation of Notch signaling pathway (GO:0045747)	6	33	0,00014146	0,009328936	78,29216362	KAT2B;CREBBP;ZMIZ1;BMP2K;EP300;RBPJ
cellular response to fluid shear stress (GO:0071498)	4	12	0,000157901	0,009778066	173,4340177	MEF2C;MAP2K5;MTSS1;NFE2L2
histone modification (GO:0016570)	10	98	0,000159976	0,009778066	39,68042673	HDAC4;CREBBP;TBL1XR1;KAT6A;MIER1;EYA4;EHMT1;EP300;TBL1X;HDAC9
activation of MAPK activity (GO:0000187)	11	117	0,000159152	0,009778066	36,29895266	ERN1;MDFIC;TAOK1;MAP3K20;S1PR2;MAPK14;TLR4;MAP2K5;DUSP6;MAP3K5;DUSP7
small GTPase mediated signal transduction (GO:0007264)	10	99	0,000174078	0,010386672	38,85336478	RB1;RAP1GDS1;ARHGAP18;PLD1;RGL1;MAPK14;RIN2;RREB1;RAPGEF6;RASGRP3
response to chemokine (GO:1990868)	3	6	0,00028676	0,014665724	322,6511655	RIPOR2;LRCH1;DOCK8
cellular response to chemokine (GO:1990869)	3	6	0,00028676	0,014665724	322,6511655	RIPOR2;LRCH1;DOCK8
negative regulation of protein localization to membrane (GO:1905476)	5	24	0,000264474	0,014665724	86,0289975	DAB2;PID1;CLTC;NUMB;RHOQ
regulation of cellular senescence (GO:2000772)	5	24	0,000264474	0,014665724	86,0289975	MAP3K3;NUAK1;CDK6;NEK6;AKT3
regulation of cell-matrix adhesion (GO:0001952)	7	52	0,000281702	0,014665724	50,60190616	MACF1;CDK6;ROCK2;PLEKHA2;RASA1;CASK;CD36
toll-like receptor signaling pathway (GO:0002224)	9	86	0,000279373	0,014665724	38,1539129	COLEC12;RPS6KA3;CD180;RFTN1;CD36;PIK3AP1;TLR4;CTSB;LGMN
protein dephosphorylation (GO:0006470)	11	125	0,000284945	0,014665724	31,49090675	PPP3CA;PTPN1;PPP1R12A;DUSP3;UBASH3B;EYA4;PTPRJ;SSH2;DUSP6;MTM1;DUSP7
clathrin coat assembly (GO:0048268)	4	14	0,00030695	0,014872154	128,198012	HIP1;CLTC;FCHO2;EPS15
stress-activated protein kinase signaling cascade (GO:0031098)	8	70	0,000331529	0,014872154	41,18596556	MAP3K3;TAOK1;MAP3K20;MAP2K5;MAP4K3;MAP3K5;STK3;MAP4K4
positive regulation of protein complex assembly (GO:0031334)	9	88	0,000332303	0,014872154	36,39576501	AIM2;PLEK;MITF;ASAP1;IDE;CD36;FNIP1;TLR4;FNIP2
negative regulation of response to stimulus (GO:0048585)	11	127	0,000327019	0,014872154	30,42268702	CSF1R;PTPN1;MAP3K3;CDK6;MCTP1;UBASH3B;VPS13C;AKT3;CASK;MERTK;MAP2K5
peptidyl-serine modification (GO:0018209)	13	170	0,000332339	0,014872154	26,56487667	SMG1;CAMK2D;PRKCH;PRKCB;ROCK2;NEK6;VRK2;MAPK14;RPS6KA3;AKT3;STK38;SGK3;CSNK1G1
positive regulation of GTPase activity (GO:0043547)	14	192	0,000324171	0,014872154	25,33656751	BCAS3;RABGAP1L;RABGAP1;DOCK8;RAP1GDS1;SNX13;DOCK10;DOCK11;TBC1D5;SRGAP2;TBC1D22A;EVI5; RAPGEF6;LIMS1
endocytosis (GO:0006897)	17	263	0,000325898	0,014872154	22,30730074	COLEC12;MSR1;DENND1B;ANXA3;DENND1A;CLTC;SNX10;DNAJC13;VRK2;SNX30;MERTK;MYO1E;STAB1;AP OE;CD36;RHOQ;CSNK1G1
cell morphogenesis involved in differentiation (GO:0000904)	7	54	0,000357359	0,015562334	47,03390656	RB1;NRP1;MEF2C;FER;OPHN1;PEAK1;SRGAP2
positive regulation of nucleic acid-templated transcription (GO:1903508)	26	502	0,000360182	0,015562334	17,53374651	RB1;HDAC4;TNNI2;EPC1;EP300;TBL1X;RREB1;MAP3K5;BPTF;ATF7IP;MEF2C;CREBBP;ZFHX3;MITF;ARID1A;T RERF1;FLI1;PIAS1;RUNX1;DAB2;MDFIC;KLHL6;TBL1XR1;KAT6A;TCF4;NFE2L2
positive regulation of protein kinase activity (GO:0045860)	13	172	0,000372103	0,015804911	25,85792583	CSF1R;PTPN1;MAP3K3;DDX3X;PRKAG2;STK3;ERN1;GPRC5B;TAOK1;LCP2;MAP2K5;MAP4K3;MAP4K4
protein localization to plasma membrane (GO:0072659)	11	130	0,00039986	0,016700803	28,90817467	NSF;MACF1;RAB40C;PACS1;ROCK2;DENND4C;FCHO2;MYO5A;FAM126A;RAPGEF6;CLASP2
filopodium assembly (GO:0046847)	4	15	0,000410397	0,016859923	112,353231	FGD3;FGD4;S1PR2;SRGAP2
substrate-dependent cell migration, cell extension (GO:0006930)	3	7	0,000492587	0,018424221	225,926277	P2RY12;NRP1;OPHN1
regulation of myosin-light-chain-phosphatase activity (GO:0035507)	3	7	0,000492587	0,018424221	225,926277	NUAK1;PPP1R12A;ROCK2
endocytic recycling (GO:0032456)	5	27	0,000472463	0,018424221	69,05407818	DENND1B;EHD4;RAB14;DENND1A;EPS15
positive regulation of epithelial cell migration (GO:0010634)	8	74	0,000485554	0,018424221	36,83959373	NRP1;BCAS3;ANXA3;ROCK2;RREB1;SASH1;FOXP1;CLASP2
positive regulation of MAP kinase activity (GO:0043406)	13	177	0,000489604	0,018424221	24,19206418	KIDINS220;MAPK14;DUSP6;STK3;DUSP7;TAOK1;PDGFC;MAP3K20;S1PR2;TLR4;MAP2K5;SASH1;MAP3K5
Ras protein signal transduction (GO:0007265)	15	223	0,000475535	0,018424221	22,13463081	RB1;CYFIP1;MYO9B;PLD1;MAPK14;EPS8;RAP2B;RAB40C;RAB14;CDC42EP3;ARHGEF3;ELMO1;RREB1;RAPGE F6;RHOQ
positive regulation of macrophage derived foam cell differentiation (GO:0010744)	4	16	0,000536523	0,019772441	99,44619823	MSR1;PRKCH;CD36;PF4
regulation of actin polymerization or depolymerization (GO:0008064)	5	28	0,000563553	0,020261222	64,52767495	EPS8;FCHSD2;RASA1;ARHGAP18;SSH2
positive regulation of macromolecule metabolic process (GO:0010604)	17	276	0,000565956	0,020261222	19,71780707	PRKN;NSF;PTPN1;MEF2C;PID1;DDX3X;ROCK2;MITF;MAPK14;P2RX7;TBC1D5;CD36;TLR4;MAP2K5;LIMS1;PF4 ;NFE2L2
MAPK cascade (GO:0000165)	17	278	0,000613884	0,021667506	19,351973	MEF2A;MAP3K3;MEF2C;CAMK2D;RANBP9;RASAL2;DUSP6;DUSP7;RASGRP3;SPRED1;RASA3;PTPRA;RASA1; MAP3K20;MAP4K3;MAP4K4;MAP3K5
regulation of B cell receptor signaling pathway (GO:0050855)	4	17	0,000687933	0,02361588	88,76169225	PRKCH;PRKCB;LPXN;RUNX1
positive regulation of transcription of Notch receptor target (GO:0007221)	4	17	0,000687933	0,02361588	88,76169225	KAT2B;CREBBP;EP300;RBPJ
regulation of primary metabolic process (GO:0080090)	11	139	0,000704639	0,023862521	24,91790643	NCOA2;CPT1A;CREBBP;TBL1XR1;CHD9;ROCK2;PLIN2;APOE;CD36;TBL1X;RGL1
positive regulation of programmed cell death (GO:0043068)	16	257	0,000726175	0,024263941	19,25692261	VAV3;DDX3X;ARHGEF12;PLEKHG5;ITSN1;ANO6;DUSP6;STK3;FGD3;AKAP13;FGD4;BCL6;ARHGEF3;MAP3K2C ;RBM5;MAP3K5
peptidyl-lysine acetylation (GO:0018394)	3	8	0,00077364	0,024541033	170,0187195	KAT2B;CREBBP;EP300
interleukin-1 beta secretion (GO:0050702)	3	8	0,00077364	0,024541033	170,0187195	AIM2;CD36;TLR4
actin cytoskeleton reorganization (GO:0031532)	7	61	0,00075858	0,024541033	37,04120254	NRP1;PTPN1;FER;PLEK;NEDD9;S1PR2;FRY
regulation of signal transduction (GO:0009966)	15	233	0,000749228	0,024541033	19,8541461	CSF1R;PTPN1;CREBBP;LIFR;MERTK;DUSP6;DHRS3;RUNX1;RGS2;UBASH3B;MDFIC;BIRC6;CD36;OGT;SASH1

activation of JUN kinase activity (GO:0007257)	5	30	0,000784503	0,024574543	56,73475523	ERN1;MDFIC;TAOK1;MAP3K20;MAP3K5
cellular protein catabolic process (GO:0044257)	7	62	0,000837165	0,025584574	35,86688257	PRKN;CLTC;AFG1L;IDE;CTSC;CTSB;LGMN
negative regulation of gene expression (GO:0010629)	29	618	0,000830448	0,025584574	14,14606109	RB1;HDAC4;PRKN;DDX3X;EHMT1;IKZF1;RBPJ;HDAC9;ELK3;NIPBL;BTAF1;ATXN1;EPC1;XDH;ATF7IP;MSR1;TSC22D4;MEF2C;DNMT3A;WWP1;ARID5B;FOXP1;ERN1;DAB2;AGO3;BCL6;MDFIC;KAT6A;LIMS1
regulation of mitotic cell cycle (GO:0007346)	12	165	0,000869403	0,026249686	22,10016466	RB1;MAP3K3;CDK19;DUSP3;NEK6;TAOK1;NEK7;CLTC;MAP2K5;MAP4K3;STK3;MAP4K4
osteoclast differentiation (GO:0030316)	4	19	0,001077157	0,031027075	72,18250344	CSF1R;SNX10;MAPK14;FOXP1
regulation of platelet activation (GO:0010543)	4	19	0,001077157	0,031027075	72,18250344	TEC;PLEK;APOE;TLR4
protein deacetylation (GO:0006476)	5	32	0,001064346	0,031027075	50,28579588	HDAC4;TBL1XR1;MIER1;TBL1X;HDAC9
plasma membrane bounded cell projection assembly (GO:0120031)	15	241	0,001055333	0,031027075	18,23213135	P2RY12;CYFIP1;NRP1;SNX10;ANO6;ASAP1;FNBP1L;MTSS1;FGD3;P2RX7;FGD4;OPHN1;S1PR2;SRGAP2;RAPGEF6
interleukin-1 beta production (GO:0032611)	3	9	0,001139137	0,031541307	134,0237202	AIM2;CD36;TLR4
interleukin-1 secretion (GO:0050701)	3	9	0,001139137	0,031541307	134,0237202	AIM2;CD36;TLR4
B cell homeostasis (GO:0001782)	3	9	0,001139137	0,031541307	134,0237202	DOCK10;DOCK11;MEF2C
pattern recognition receptor signaling pathway (GO:0002221)	6	48	0,001145355	0,031541307	38,42491534	RPS6KA3;CD180;CD36;TLR4;CTSB;LGMN
activation of MAPKK activity (GO:0000186)	5	33	0,001229147	0,033480906	47,46768223	KIDINS220;TAOK1;MAP3K20;STK3;MAP3K5
regulation of transcription, DNA-templated (GO:0006355)	59	1598	0,001359434	0,036631638	10,40275309	RB1;ZMYND8;EHMT1;JMJD1C;IKZF1;RBPJ;NR3C1;BACH1;ELK3;RPS6KA3;NIPBL;NFKBIZ;EPC1;EP300;MAP3K5;NCOA2;TSC22D4;MEF2C;PRKCB;TCF12;ARID5B;MITF;ETV1;ARID1A;PIAS1;FOXP1;RUNX1;MDFIC;TBL1XR1;KAT6A;IRF2;ATF7;HDAC4;CAMK2D;BAZ2B;HDAC9;PHF21A;BTAF1;ATXN1;CUX1;TNNI2;APOE;TBL1X;RREB1;BPTF;ATF7IP;CREBBP;ZFHX3;WWP1;MAPK14;TRERF1;FLI1;DAB2;BCL6;KLHL6;TCF4;OGT;LIMS1;NFE2L2
regulation of bone mineralization (GO:0030500)	6	50	0,001422647	0,037527938	35,50043315	P2RX7;MEF2C;NBR1;BMP2K;ANO6;SLC8A1
positive regulation of JUN kinase activity (GO:0043507)	6	50	0,001422647	0,037527938	35,50043315	ERN1;MDFIC;TAOK1;MAP3K20;SASH1;MAP3K5
protein autophosphorylation (GO:0046777)	12	175	0,001445821	0,03774194	19,23728331	ERN1;CSF1R;MAP3K3;FER;SMG1;CAMK2D;TEC;PEAK1;NEK6;TAOK1;ULK2;VRK2
regulation of B cell apoptotic process (GO:0002902)	3	10	0,001597474	0,041270823	109,1402338	BCL6;FNIP1;FOXP1
lipid phosphorylation (GO:0046834)	5	35	0,001614282	0,041279497	42,49684898	SMG1;DGKD;PIK3CA;DGKZ;FAM126A
SRP-dependent cotranslational protein targeting to membrane (GO:0006614)	8	89	0,001648276	0,041723023	25,18985609	RPS28;RPS27;RPS29;RPL37A;RPLP2;RPL38;RPL37;RPS21
cellular response to insulin stimulus (GO:0032869)	9	110	0,001674549	0,041964208	22,69405858	ATP6V1A;KAT2B;FER;DENND4C;MYO5A;IDE;HDAC9;RHOQ;ATP6V0A1
positive regulation of endothelial cell migration (GO:0010595)	7	70	0,001722419	0,042317468	28,11247957	NRP1;BCAS3;ANXA3;ROCK2;AKT3;SASH1;FOXP1
regulation of Notch signaling pathway (GO:0008593)	7	70	0,001722419	0,042317468	28,11247957	KAT2B;CREBBP;ZMIZ1;FBXW7;BMP2K;EP300;RBPJ
positive regulation of cell-substrate adhesion (GO:0010811)	6	52	0,00174836	0,042537775	32,88562705	NRP1;CDK6;PLEKHA2;CD36;RREB1;LIMS1
protein acylation (GO:0043543)	5	36	0,001836973	0,043842411	40,29722761	KAT2B;CREBBP;ZDHHC20;KAT6A;EP300
histone deacetylation (GO:0016575)	5	36	0,001836973	0,043842411	40,29722761	HDAC4;TBL1XR1;MIER1;TBL1X;HDAC9
response to insulin (GO:0032868)	7	71	0,001870815	0,044228899	27,31244616	KAT2B;FER;DENND4C;MYO5A;HDAC9;OGT;RHOQ
nuclear-transcribed mRNA catabolic process, nonsense-mediated decay (GO:0000184)	9	112	0,00189756	0,044441913	21,81589639	RPS28;SMG1;RPS27;RPS29;RPL37A;RPLP2;RPL38;RPL37;RPS21
positive regulation of signal transduction (GO:0009967)	13	206	0,001969722	0,045704856	16,77730297	MACF1;CREBBP;PRKCB;CD180;LY86;RBPJ;ERN1;KAT2B;ZMIZ1;ASXL2;BMP2K;EP300;SASH1
regulation of organelle organization (GO:0033043)	9	113	0,00201763	0,046386988	21,39350392	PRKN;RABGAP1L;ZEB2;RABGAP1;TBC1D5;TAOK1;TBC1D22A;EVI5;MTM1
relaxation of cardiac muscle (GO:0055119)	3	11	0,002156266	0,047819503	91,04459526	CAMK2D;RGS2;SLC8A1
regulation of hemopoiesis (GO:1903706)	3	11	0,002156266	0,047819503	91,04459526	CREBBP;PRKCB;RUNX1
negative regulation of cellular senescence (GO:2000773)	3	11	0,002156266	0,047819503	91,04459526	MAP3K3;CDK6;AKT3
relaxation of muscle (GO:0090075)	3	11	0,002156266	0,047819503	91,04459526	CAMK2D;RGS2;SLC8A1
cotranslational protein targeting to membrane (GO:0006613)	8	93	0,002181607	0,047957081	22,94960078	RPS28;RPS27;RPS29;RPL37A;RPLP2;RPL38;RPL37;RPS21
regulation of protein oligomerization (GO:0032459)	4	23	0,002275228	0,049152761	50,74000063	AIM2;EP300;IDE;APOE
regulation of apoptotic process (GO:0042981)	34	815	0,00226854	0,049152761	10,74187627	PRKN;CSF1R;HIP1;DDX3X;ITSN1;STK3;FGD3;FGD4;RPS6KA3;AKAP13;MAP3K20;MAP2K5;MAP4K3;RBM5;MAP3K5;CTSB;MAP4K4;VAV3;MAP3K3;MEF2C;CREBBP;ARHGEF12;PLEKHG5;ANO6;MERTK;DUSP6;RNF144B;DAB2;BCL6;TAOK1;RASA1;ARHGEF3;BIRC6;SGK3

Table S2 (related to Figure 6 and Figure S6): Enriched Gene Ontology (GO) ‘Biological Process’ terms for upregulated differentially expressed genes.

Results from Gene Ontology (GO) enrichment analysis of ‘Biological Process’ based on scRNA-seq data from synovial myeloid cells ($CD45^{+}CD11b^{+}$) from mice pre-treated with β-glucan or PBS and 7 days later subjected to K/BxN-STA for 17 days. Enrichment analysis was performed with upregulated differentially expressed genes (ranked according to Log_2 fold change) in the main myeloid cell compartment comprising the clusters 1-8 and 13 (see Figure 6A). Significantly enriched GO terms were defined by FDR < 0.05. ‘Term’ is the functional annotation in the database; ‘In.List’ is the number of genes in the list of interest with this functional annotation; ‘In.Annotation’ is the total number of genes with this functional annotation; ‘p_val’ is the uncorrected p-value; ‘p_val_adj’ is the p-value adjusted for multiple testing; ‘Combined.Score’ is the enrichment score as reported by enrichr; ‘Genes’ are the genes in the differentially expressed gene list that are contained in the functional annotation.
